# Assessing the impact of a national social marketing campaign for antimicrobial resistance on public awareness, attitudes, and behaviour, and as a supportive tool for healthcare professionals, England, 2017 to 2019

**DOI:** 10.2807/1560-7917.ES.2023.28.47.2300100

**Published:** 2023-11-23

**Authors:** Ellie L Gilham, Ella Casale, Alison Hardy, Adeola H Ayeni, Ella Sunyer, Tori Harris, Rachel Feechan, Anna Heltmann, Malcolm Fawcett, Susan Hopkins, Diane Ashiru-Oredope

**Affiliations:** 1 HCAI, Fungal, AMR, AMU and Sepsis Division, the United Kingdom Health Security Agency, London, United Kingdom; 2 Behavioural Programmes Unit, Office of Health Improvement and Disparity (OHID), London, United Kingdom; 3 Kantar Public, London, United Kingdom; 4 Teesside University, Middlesbrough, United Kingdom

**Keywords:** behaviour change, public campaign, antibiotic resistance, health inequalities, primary care physicians

## Abstract

**Background:**

Previous United Kingdom campaigns targeting antimicrobial resistance (AMR) recommended running multimedia campaigns over an increased timeframe. The 3-year-long Keep Antibiotics Working (KAW) campaign was a mass media campaign in England targeting the public and general practitioners (GPs).

**Methods:**

Every year, pre- and post-campaign questionnaire data were collected from the public, whereas post-campaign interview data were obtained from GPs. Data were weighted to allow pre- and post-campaign comparisons between independent samples. Significant changes in nominal and ordinal data were determined using Pearson’s chi-squared (*X* ^2^) and Mann–Whitney U tests, respectively.

**Results:**

Prompted campaign recognition was high, increasing by 6% from 2018 to 2019 (2017: data unavailable; 2018: 68% (680/1,000); 2019: 74% (740/1,000); *X* ^2^ = 8.742, p = 0.003). Knowledge regarding declining antibiotic effectiveness when taken inappropriately improved following the campaign (net true: pre-2017 = 69.1% (691/1,000); post-2019 = 77.6%; (776/1,000); *X* ^2^ = 5.753, p = 0.016). The proportion of individuals reporting concern for themselves or for children (≤ 16 years) about AMR increased by 11.2% (*Z* = −5.091, p < 0.001) and 6.0% (*Z* = −3.616, p < 0.001) respectively, pre- to post-campaign. Finally, in 2017, reported confidence to say no to patients requesting antibiotics differed significantly between GPs who were and were not aware of the campaign (net agree: 98.9% (182/184) vs 92.4% (97/105) respectively; *X* ^2^ = 4.000, p = 0.045).

**Conclusion:**

A high level of prompted campaign recognition was achieved. The KAW campaign improved aspects of AMR knowledge and certain attitudes towards appropriate antimicrobial use. It increased awareness of and concern about AMR, supporting GP confidence to appropriately prescribe antibiotics. Future determination of measurable behaviour changes resulting from AMR campaigns is important.

**Key public health message**

**What did you want to address in this study?**

We studied the impact of a national public health campaign in England on antimicrobial resistance and correct antibiotic usage. We assessed the public’s campaign recognition and if knowledge, awareness and understanding of antimicrobial resistance improved. We also checked if reports of correct antibiotic usage increased, if general practitioners felt more confident to decline antibiotics and if patients’ expectations for antibiotics reduced.

**What have we learnt from this study?**

We learnt that a campaign on antimicrobial resistance which uses marketing to encourage behaviour change that is beneficial to society (social marketing) and which is promoted via multiple channels, including television, social media and patient resources (e.g. information leaflets and posters) helps to improve public knowledge on its key messages and supports general practitioners’ confidence to prescribe antibiotics appropriately.

**What are the implications of your findings for public health?**

Our findings suggest that campaigns using a multifactorial, social marketing approach may increase public knowledge and concern about antimicrobial resistance. However, whether campaigns actually lead to reduced antibiotic use and decreases in antimicrobial-resistant infections needs to be further investigated with outcomes that we can measure, such as events of inappropriate prescribing or occurrences of antimicrobial resistant infections.

## Introduction

Antimicrobial resistance (AMR) is a considerable threat to human health. Globally, an estimated 1.2 million people died in 2019 from antibiotic resistant bacterial infections [1]. The widely referenced AMR Review report by O’Neill et al. estimated that by 2050 drug-resistant infections could kill ca 10 million people globally each year, costing the world economy $100 trillion annually [2]. One of the main recommendations from the AMR Review report was the need to develop “*a massive global public awareness campaign on AMR*”.

Previous United Kingdom campaigns, which aimed to optimise prescribing and reduce public demand for antibiotics, used simple single-channel approaches, such as distributing posters or leaflets to healthcare practices over short time periods [3,4]. Evaluations of these campaigns recommended the importance of running mass multimedia campaigns over longer periods [5]. Social marketing is defined as the application of commercial marketing techniques to the analysis, planning, execution, and evaluation of programmes created to influence the voluntary behaviour of target audiences to improve their personal welfare and that of society [6]. Social marketing is an effective tool to change behaviour to facilitate the prevention of communicable disease, for example though the promotion of infection prevention control behaviours [7]. Therefore, Keep Antibiotics Working (KAW) was developed as England’s first multi-channel, integrated social marketing and communications campaign targeting the public and supporting general practitioners (GPs) in prudent antibiotic prescribing. This campaign was designed as part of an integrated policy to support public behaviour change. Other initiatives in the integrated policy included eBug, a free educational resource for 3–16-year-olds [8], Antibiotic Guardian, a pledge-based behaviour change tool [9], and the Help Us Help You winter campaign [10]. However, the population groups that were targeted [8,9] and the campaign messages used [10] differed from KAW.

Market research with prescribers and the public to investigate pre-campaign knowledge, attitudes and behaviour towards AMR and antibiotic prescribing had identified that the public had limited understanding of AMR and misconceptions, for example that antibiotics are effective against viral infections, were common [11-13]. Furthermore, individuals who recognised AMR as an issue perceived this to be a global problem to be tackled by the scientific community and not something their actions could positively affect. For patients who recognised that antibiotics were not always necessary, the likelihood of them requesting antibiotics from their GP depended on their health status which influenced whether the patient used ‘cold state’ or ‘hot state’ cognition [14].

National surveillance data from England in 2015 estimated that most antibiotics across England are prescribed in general practice (74%) [15]. If during patient-GP interaction, the patient expects antibiotics, the GP, where limited by time, can feel pressurised to prescribe. If antibiotics are prescribed, the patient may credit them for their recovery, even though they might have recovered without an antibiotic. This may reinforce the behaviour, normalising the expectation for an antibiotic prescription [15,16]. Therefore, messaging aimed to reduce patient expectation for an antibiotic prescription may support confidence of GPs to prescribe as appropriate. In addition to the general public, prior evidence suggested that key audiences to achieve high levels of campaign recognition should be mothers of children aged 0–16 years as they are likely to have primary responsibility for their child(ren)’s health, and men and women aged over 50 years [17]. These insights guided the development of the campaign, which aimed to raise public awareness and understanding of AMR, and to reduce unnecessary demand for antibiotics.

The aim of the current study was to evaluate the national KAW campaign and in particular to assess whether it reached the target audiences in England, improved knowledge, awareness and understanding of AMR, increased reported action of appropriate antibiotic usage behaviours, strengthened GP confidence to decline antibiotics and reduced patient expectation for antibiotics.

## Methods

### Development of the campaign

Development of the KAW campaign followed the Government Communication Service's Objectives, Audience/insight, Strategy/idea, Implementation and Scoring (OASIS) model and the Wellcome Trust's key principles for communicating AMR effectively [18,19].

Between February and April 2017, a pilot campaign was run through an Independent Television (ITV) called Granada Television, which broadcasts to all individuals living within Yorkshire and the North-West region of England. This pilot campaign was then evaluated to inform and support the development of the national KAW campaign.

### Campaign summary

The national KAW campaign then ran for three 3-month periods between November and January 2017, 2018, and 2019 using broadcast advertising (a television (TV) commercial, video on demand via YouTube, radio, newspaper advertising and posters) and advertising via social (Facebook, Twitter, and Instagram) and news media. People who were searching online for information on cold and influenza were served with an advert encouraging them to go to the pharmacy instead of the GP to promote self-care for mild colds and influenza virus infections. The direct-to-public communications were supplemented with posters, leaflets and ‘Treat Your Infection’ non-prescription pads providing advice on how respiratory tract and urinary tract infections can be managed at home if antibiotics are not required. In the first year of the campaign, these materials along with a letter outlining where to order additional resources, were distributed to all GP practices in England. The posters were also distributed to all community pharmacies in England. Illustrative examples of posters are provided in Supplementary Images 1 and 2. In the second and third years of the campaign, healthcare professionals could download from the Public Health England Campaign Resource Centre and customise for their surgeries. These resources were mostly aimed at primary care providers.

In addition to an overall evaluation of the campaign to determine its effectiveness and share learning with other countries, year-on-year optimisation was attempted to continuously improve outcomes towards the campaign aim. At each step, an informal evaluation was conducted followed by adaption of the channel mix and creative solutions.

### Data collection

Data collection from the public in England occurred in six steps; one pre and one post each wave of advertising in 2017, 2018 and 2019. Interviews with the public were conducted by Kantar Public, using a Computer Assisted Web Interviewing (CAWI) approach, with samples drawn from the Kantar Profiles (Lightspeed) Online Panel Network, an industry-leading double opt-in panel, built with highly validated and trusted sources and partners. Questionnaires were adjusted every year to account for changes to the campaign, updated audiences and messaging, and to incorporate learning or fill gaps from previous evaluations. A seven-point Likert scale, where 1 represented no concern at all and 7 represented a high level of concern, was used to determine the level of concern individuals felt regarding AMR. The CAWI questionnaire is available in Supplementary Material 2. Key subgroups included mothers of children aged 0–16 years and adults aged over 50 years.

The target sample size for questionnaire responses from the public was 1,000 respondents in each wave, providing a sufficiently large base size to allow robust analysis both of the overall sample, and of key subgroups. Each year, purposive sampling was conducted among mothers of children aged 0–16 years to increase the sample size of this sub-group and therefore allow for sub-group analyses.

Three waves of quantitative research with GPs (following each year of the campaign) involved a target sample size of 300 participants to ensure a sufficient sample size. Interviews with GPs were conducted via telephone using Computer Assisted Telephone Interviewing (CATI) with a pre-defined questionnaire, which is presented in Supplementary Material 3. Samples were drawn from the GP practices’ database available on the National Health Service (NHS) Digital website. The GP sample was purchased from a specialist health professional database and quotas were set on GP practice size and region to ensure the sample was representative across regions and practice size.

A count of video (accessible via a weblink available in Supplementary Material 1) views was automatically generated every time the video was played on social media; this count was supplied by a media agency (Wavemaker). Media coverage was supplied by a Public Relations agency (Freuds) and included a count of mentions in a newspaper article or the news. Poster distribution data were supplied by the Resource Centre. Response levels and cost per click for the online campaign resources were monitored in real-time, to assess metrics of engagement with advertising and identify the best-performing adverts in the population.

### Statistical analysis

The public samples (overall samples) collected at each campaign wave were weighted for the statistical analysis to ensure the samples were matched and nationally representative on the demographic variables of age (sample restricted to over 18-year-olds), sex (collected as a binary variable), region, and socioeconomic status (SES), allowing for pre- post-campaign comparisons to be made.

For GPs, comparisons between individuals who were and were not aware of the campaign were made to determine any significant differences between the two groups. This is due to data only being collected from GPs following each year of the campaign. 

Pearson’s chi-squared, Pearson’s cumulative test statistic (χ^2^), or Mann–Whitney U tests, with the Z-score, were used to outline significant changes in nominal and ordinal data respectively for pre- and post-campaign measures of attitudes, knowledge, and concern regarding AMR. Tests were initially conducted to determine the effect of the campaign from pre-2017 to post-2019; tests were then conducted to outline the significance of year-by-year changes. Questionnaire response categories for questions assessing attitudes, knowledge, and reported behaviour (such as those further presented in tables 5 and 6) were pooled before statistical testing to give two response categories as opposed to four (for example, ‘true’ or ‘false’ as opposed to ‘definitely true’, ‘probably true’, ‘probably false’ and ‘definitely false’). A p value threshold of p < 0.05 was used to determine significance. Data were analysed using Microsoft Excel and IBM SPSS Statistics Version 27.0.

## Results

### Participant demographics

Participant demographics are shown in Table 1. Mean age and standard deviation within each sample was similar across the campaign (pre-2017: 46.9 ± 16.4 years; post-2017: 47.8 ± 16.6 years; pre-2018: 46.4 ± 15.9 years; post-2018: 47.2 ± 15.6 years; pre-2019: 47.9 ± 15.5 years; post-2019: 45.8 ± 16.3 years). There was a slightly higher proportion of females within each sample. Black ethnicities, including African and Caribbean, had the lowest proportion of respondents at each data collection point. Finally, participants were evenly distributed between the four SES groups.

**Table 1:** Demographics of questionnaire respondents at each data collection point across the 3-year KAW campaign, England, 2017–2019

Demographic characteristics		Pre 2017 (n = 1,000)		Post 2017 (n = 1,201)		Pre 2018 (n = 1,350)		Post 2018 (n = 1,352)		Pre 2019 (n = 1,572)		Post 2019 (n = 1,350)	
		Number	%	Number	%	Number	%	Number	%	Number	%	Number	%
Sex^a^	Female	510	51.0	610	50.8	759	56.2	788	58.3	865	55.0	767	56.8
Ethnicity	British	853	85.3	1,034	86.1	1,162	86.1	1,153	85.3	1,347	85.7	1,133	83.9
	Other white background	72	7.2	61	5.1	80	5.9	68	5.0	83	5.3	81	6.0
	Mixed background	13	1.3	30	2.5	26	1.9	27	2.0	36	2.3	35	2.6
	Asian	35	3.5	42	3.5	47	3.5	72	5.3	60	3.8	60	4.4
	Black African or Caribbean	12	1.2	15	1.2	17	1.3	20	1.5	27	1.7	22	1.6
	Other	7	0.7	6	0.5	9	0.7	4	0.3	9	0.6	8	0.6
	Prefer not to say	8	0.8	12	1.0	9	0.7	8	0.6	10	0.6	11	0.8
	Missing	0	0	1	0.1	0	0	0	0	0	0	0	0
Socioeconomic status	AB	281	28.1	302	25.1	368	27.3	356	26.3	437	27.8	375	27.8
	C1	274	27.4	340	28.3	372	27.6	362	26.8	407	25.9	345	25.6
	C2	210	21.0	200	16.7	226	16.7	236	17.5	261	16.6	218	16.1
	DE	235	23.5	359	29.9	384	28.4	398	29.4	467	29.7	412	30.5

AB: higher and intermediate managerial, administrative, professional occupations; C1: supervisory, clerical, and junior managerial, administrative, professional occupations; C2: skilled manual occupations; DE: semi-skilled and unskilled manual occupations, unemployed and lowest grade occupations; KAW: Keep Antibiotics Working.

^a^ Sex was collected as a binary variable (male/female) and there were no missing data on sex among study participants.

Each year the questionnaire respondents were anonymous, so whether some people participated in the study at more than one collection point is unknown.

### Campaign reach

The 2017/18 campaign received over 10 million views on social media, the highest across all three campaign years (Table 2). Despite the lowest spend (£1.5 million; €1.7 million), the 2019/20 campaign received the second highest number of views (5.6 million). Attitudes towards the campaign itself were mostly positive. Following the final year of the campaign, most participants continued to believe the adverts were clear (net agree: 85% (850/1,000)) and important (net agree: 83.6% (836/1,000)). However, the proportion of participants who were ‘fed up with seeing this type of advertising’ appeared to increase slightly (2017: 14.2% (142/1,000); 2019: 17.9% (179/1,000)), suggesting some campaign fatigue may have started developing.

**Table 2:** Video views on social media (n = 18.4 million), coverage within news articles (n = 1,146) and number of leaflets, posters and non-prescription pads distributed to healthcare settings (n = 120,986) across the 3-year KAW campaign, England, 2017–2019

Campaign	Budget (million)	Number of video views (million)	Coverage^a^ (pieces)	Number of leaflets and posters distributed	Number of non-prescription pads^b^ distributed
2017/18	£3, €3.4	10.3	769	629,420	47,405
2018/19	£2, €2.6	2.5	283	399,096	36,295
2019/20	£1.5, €1.7	5.6	94	2,350,592	37,286

KAW: Keep Antibiotics Working.

^a^ Coverage consisted of the count of mentions of the campaign in newspaper articles or the news.

^b^ Non-prescription pads were physically distributed in 2017 while in 2018 resources became digital and could be downloaded from the campaign resource centre. Non-prescription pads for respiratory tract infections were introduced in 2017, and for urinary tract infections in 2018.

### Campaign recognition

There was a significant difference in unprompted recognition (participant recall of a campaign without help of suggestions from interviewers) of any AMR publicity by the public and the key subgroups from before the 2017 to following the 2019 campaign (all participants, *X^2^* = 52.263, p < 0.001; mothers, *X^2^* = 8.919, p = 0.003; adults aged over 50 years, *X^2^* = 19.632, p < 0.001) (Table 3). Unprompted recognition was significantly lower in adults aged over 50 years following the campaign in 2018 (*X^2^* = 10.971, p < 0.001) and 2019 (*X^2^* = 13.851, p < 0.001) compared with the public.

**Table 3:** Results of surveying the public and campaign target groups to assess their unprompted^a^ or prompted^b^ recognition of the KAW campaign**,** England, 2017–2019

People surveyed and responses		Pre 2017		Post 2017		Pre 2018		Post 2018		Pre 2019		Post 2019	
		n	%	n	%	n	%	n	%	n	%	n	%
Unprompted^a^ recognition of any AMR publicity													
All*^,c^	Yes	126	12.6	335*^,c^	33.5	148	14.8	243*^c^	24.3	171	17.1	252*^,c^	25.2
	No	807	80.7	592*^,c^	59.2	773	77.3	682*^,c^	68.2	755	75.5	678*^,c^	67.8
	Don’t know	67	6.7	74	7.4	79	7.9	75	7.5	75	7.5	70	7.0
	**Total**	**1,000**	**100**	**1,001**	**100**	**1,000**	**100**	**1,000**	**100**	**1,001**	**100**	**1,000**	**100**
Mothers*^,c,d^	Yes	21	16.9	51*^,c^	38.9	20	15.3	37*^,c^	28.5	28	21.4	41	31.3
	No	90	72.6	80*^,c^	61.1	100	76.3	83*^,c^	63.8	91	69.5	81	61.8
	Don’t know	13	10.5	0	0.0	11	8.4	10	7.7	12	9.2	9	6.9
	**Total**	**124**	**100**	**131**	**100**	**131**	**100**	**130**	**100**	**131**	**100**	**131**	**100**
Adults aged over 50 years*^,c^	Yes	37	8.8	148	34.7	53	12.9	77	19.0	52	12.5	80	19.3
	No	357	84.8	245	57.2	323	78.0	329	81.0	364	87.5	335	80.7
	Don’t know	27	6.4	35	8.1	38	9.1	0	0.0	0	0.0	0	0.0
	**Total**	**421**	**100**	**428**	**100**	**414**	**100**	**406**	**100**	**416**	**100**	**415**	**100**
Prompted^b^ campaign recognition													
All*^,e^	Yes	NA	NA	NC	NA	NA	NA	680	68.0	NA	NA	740	74.0
	No	NA	NA	NC	NA	NA	NA	320	32.0	NA	NA	260	26.0
	**Total**	**NA**	**NA**	**NC**	**NA**	**NA**	**NA**	**1,000**	**100**	**NA**	**NA**	**1,000**	**100**
Mothers*^,d,e^	Yes	NA	NA	NC	NA	NA	NA	91	69.5	NA	NA	109	83.2
	No	NA	NA	NC	NA	NA	NA	40	30.5	NA	NA	22	16.8
	**Total**	NA	NA	**NC**	**NA**	NA	NA	**131**	**100**	NA	NA	**131**	**100**
Adults aged over 50 years*^,e^	Yes	NA	NA	NC	NA	NA	NA	283	69.7	NA	NA	298	71.8
	No	NA	NA	NC	NA	NA	NA	123	30.3	NA	NA	117	28.2
	**Total**	NA	NA	**NC**	**NA**	NA	NA	**406**	**100**	NA	NA	**415**	**100**
GPs	Yes	NA	NA	184	63.7	NA	NA	178	62.0	NA	NA	205	60.1
	No	NA	NA	105	36.3	NA	NA	109	38.0	NA	NA	136	39.9
	**Total**	NA	NA	**289**	**100**	NA	NA	**287**	**100**	NA	NA	**341**	**100**

AMR: antimicrobial resistance; GP: general practitioner; KAW: Keep Antibiotics Working; n: sample size; NA: not applicable; NC: data not collected at this time point.

^a^ Unprompted recognition, participant recall of a campaign without help of suggestions from interviewers.

^b^ Prompted recognition, participant recall of a campaign with help of suggestions from interviewers, i.e. the participant is shown campaign material and asked if they recognise it.

^c^ *X^2^* and p values can be found in Supplementary Material 4a.

^d^ These were mothers of children aged 0−16 years.

^e^ *X^2^* and p values can be found in Supplementary Material 4b.

A significant difference (p < 0.05) is marked with an asterisk (*). Each year the questionnaire respondents were anonymous, so whether some people participated in the study at more than one collection point is unknown.

Prompted campaign recognition (the participant is shown campaign material and asked if they recognise it) was higher than unprompted recognition and increased significantly in the public and in mothers of children aged 0–16 years between 2018 and 2019 (*X^2^* = 8.742, p = 0.003; *X^2^* = 9.283, p = 0.002). Prompted recognition was similarly as high in adults aged over 50 years as in mothers in 2018 but did not change significantly from 2018 to 2019. Prompted recognition was significantly higher post-2019 in mothers of children aged 0–16 years compared with the public (*X^2^* = 6.769, p = 0.009). GP awareness of the campaign was lower than for the public following the campaign in 2018 and 2019. The TV campaign had the greatest recognition followed by information seen at the doctor’s surgery/clinic and newspaper articles, as shown in Supplementary Material 5.

### Changes in knowledge, awareness and understanding

Perceived knowledge (Table 4) of antibiotic resistance and AMR improved significantly following the 3-year campaign (*X^2^* = 13.952, p = 0.003 and *X^2^* = 20.219, p < 0.001 respectively). Perceived knowledge of AMR was lower than that of antibiotics before the campaign (23.4% vs. 58%) but increased to a slightly greater extent following the campaign (9.2 vs. 8.1% increase). However, despite declining consistently over the duration of the campaign, the proportion of individuals who had never heard of the term ‘antimicrobial resistance’ following the campaign remained high (40.5%, 387/956) compared with 7.6% (74/969) for antibiotic resistance. There were significant differences in perceived knowledge of AMR depending on sex, with males reporting significantly greater perceived knowledge (*X^2^* = 14.121, p = 0.003); this difference remained following the campaign (*X^2^* = 13.611, p = 0.003). Furthermore, a significant social gradient was identified for antibiotic resistance with higher SES groups reporting higher perceived knowledge (*X^2^* = 13.144, p = 0.004); the significance of this gradient reduced following the campaign (*X^2^* = 8.138, p = 0.043).

**Table 4:** Descriptive statistics of perceived knowledge of antimicrobial and antibiotic resistance pre- and post-KAW-campaign among questionnaire respondents in the public, by sex, socioeconomic status, and ethnicity, England, 2017–2019

Type of resistance and sociodemographic characteristics		Pre 2017									Post 2019								
		Know a lot		Know something		Heard of but know nothing about		Never heard of		Total	Know a lot		Know something		Heard of but know nothing about		Never heard of		Total
		n	%	n	%	n	%	n	%		n	%	n	%	n	%	n	%	
Antibiotic resistance																			
All*		**84**	**8.7**	**477**	**49.3**	**320**	**33.1**	**86**	**8.9**	**967**	**101**	**10.4**	**540**	**55.7**	**254**	**26.2**	**74**	**7.6**	**969**
Sex^a^	Male*	42	8.7	228	47.1	166	34.3	48^a^	9.9	484^a^	43	9.1	274	58.3	117	24.9	36	7.7	470
	Female	42	8.7	249	51.4	154	31.8	39^a^	8.1	484^a^	58	11.6	266	53.3	137	27.5	38	7.6	499
SES^a^	ABC1*^,b^	60^a^	11.2	271^a^	50.4	164	30.5	43	8.0	538	65	12.1	306	57.0	124	23.1	42	7.8	537
	C2DE*^,b^	23^a^	5.4	207^a^	48.3	156	36.4	43	10.0	429	36	8.3	234	54.2	130	30.1	32	7.4	432
Ethnicity^a^	White*	74	8.2	445	49.6	299^a^	33.3	79	8.8	897^a^	90^a^	10.2	493^a^	55.9	235	26.6	64^a^	7.3	882^a^
	Non-white	10	14.5	32	46.4	20^a^	29.0	7	10.1	69^a^	10^a^	12.5	43^a^	53.8	16	20.0	11^a^	13.8	80^a^
	Prefer not to say	0	0.0	0	0.0	0^a^	0.0	0	0.0	0^a^	0^a^	0.0	3^a^	50.0	3	50.0	0^a^	0.0	6^a^
Antimicrobial resistance																			
All*		**43**	**4.5**	**179**	**18.9**	**273**	**28.9**	**451**	**47.7**	**946**	**48**	**5.0**	**264**	**27.6**	**257**	**26.9**	**387**	**40.5**	**956**
Sex	Male*	29	6.1	90	19.0	153	32.3	202	42.6	474	25	5.4	151	32.5	123	35.6	165	35.6	464
	Female	14	3.0	89	18.9	120	25.4	249	52.8	472	23	4.7	113	23.0	134	27.2	222	45.1	492
SES^a^	ABC1*^,b^	27	5.1	107	20.4	156^a^	29.7	235	44.8	525^a^	35^a^	6.6	150	28.1	145	27.2	203^a^	38.1	533
	C2DE*^,b^	16	3.8	72	17.1	118^a^	28.0	216	51.2	422^a^	14^a^	3.3	114	27.0	112	26.5	183^a^	43.3	423
Ethnicity^a^	White*	39	4.4	161	18.3	252^a^	28.7	426	48.5	878^a^	40	4.6	244^a^	28.0	231	26.5	356	40.9	871^a^
	Non-white	4	5.8	18	26.1	22^a^	31.9	25	36.2	69^a^	9	11.4	19^a^	24.1	24	30.4	27	34.2	79^a^
	Prefer not to say	0	0.0	0	0.0	0^a^	0.0	0	0.0	0^a^	0	0.0	0^a^	0.0	2	40.0	3	60.0	5

KAW: Keep Antibiotics Working; n: sample size; SES: socioeconomic status.

^a^ For some of the socio-demographic characteristics, summing up numbers in corresponding columns results in slightly less or more counts than the total displayed in the ‘All’ category. These discrepancies result from weighting to allow comparisons between independent samples.

^b^ ABC1: Higher and intermediate managerial, administrative, supervisory, clerical and junior managerial and professional occupations; C2DE: skilled manual occupations, semi-skilled and unskilled manual occupations, unemployed and lowest grade occupations.

A significant difference (p < 0.05) is marked by an asterisk (*); *X* ^2^ and p values are included in Supplementary Material 6. Each year the questionnaire respondents were anonymous, so whether some people participated in the study at more than one collection point is unknown.

GPs’ perceptions of patient knowledge (assessed using a 0 to 10 scale where 0 is none of their patients were aware of AMR and 10 is all patients are aware of AMR) improved during the campaign with a ca 27% reduction in the number of GPs who thought patients were unaware of issues relating to AMR following the campaign (post-campaign 2017: 45% (135/300) scoring ≥ 6 vs 56.7% (170/300) scoring ≤ 4; post-campaign 2019: 43.8% (152/347) scoring ≥ 6 vs 29.7% (103/347) scoring ≤ 4).

Table 5 shows the public attitude and knowledge of antibiotic resistance and reported antibiotic usage behaviours. General understanding of what antibiotics should be used for was relatively strong and remained consistent throughout the campaign (net agree ‘Antibiotics don’t work for everything’, pre-2017 = 92.3% post-2019 = 89.6%). However, following the campaign, 60.9% and 48.3% of participants thought colds and influenza respectively ‘were not treated with antibiotics’. This declined significantly by 6.7 (*X^2^* = 9.772, p = 0.02) and 7.3% (*X^2^* = 10.096, p = 0.01) respectively from pre-campaign levels.

**Table 5:** Descriptive statistics to assess knowledge, attitude and behaviour towards antibiotic resistance and appropriate antibiotic usage among the general public and in parents, concerning themselves or their child respectively, across the 3-years KAW campaign, England 2017–2019

People and their standpoint or opinions		Pre 2017		Post 2017		Pre 2018		Post 2018		Pre 2019		Post 2019	
		n	(%)	n	(%)	n	(%)	n	(%)	n	(%)	n	(%)
General public													
Colds are not treated with antibiotics*^,a^		676	67.6	678	67.8	702	70.2	660	66.0	652	65.2	609	60.9
Flu^b^ is not treated with antibiotics*^,a^		556	55.6	555	55.5	560	56.0	527	52.7	552	55.2	483	48.3
Antibiotics don’t work for everything*^,a^	Strongly agree	574	57.4	633	63.3	598	59.8	628	62.9	646	64.5	601	60.1
	Agree	349	34.9	266	26.6	314	31.4	263	26.3	259	25.9	295	29.5
	Disagree	30	3.0	42	4.2	36	3.6	50	5.0	44	4.4	53	5.3
	Strongly disagree	14	1.4	16	1.6	16	1.6	17	1.7	18	1.8	19	1.9
	Don’t know	33	3.3	43	4.3	36	3.6	41	4.1	34	3.4	32	3.2
**Total number of respondents and %**		**1,000**	**100**	**1,000**	**100**	**1,000**	**100**	**999**	**100**	**1,001**	**100**	**1,000**	**100**
Antibiotics will stop working for you if taken for the wrong things*^,a^	Definitely true	225	22.5	289	28.9	302	30.2	327	32.7	336	33.6	350	35.0
	Probably true	466	46.6	472	47.2	471	47.1	447	44.7	455	45.5	426	42.6
	Probably false	144	14.4	92	9.2	99	9.9	79	7.9	101	10.1	116	11.6
	Definitely false	17	1.7	19	1.9	15	1.5	21	2.1	16	1.6	18	1.8
	Don’t know	148	14.8	127	12.7	112	11.2	125	12.5	93	9.3	91	9.1
**Total number of respondents and %**		**1,000**	**100**	**999**	**100**	**999**	**100**	**999**	**100**	**1,001**	**100**	**1,001**	**100**
Taking antibiotics when you don’t need them puts you and your family at risk of antibiotic resistant infections*^,a^	Definitely true	NC	NA	363	36.3	407	40.7	455	45.5	468	46.8	482	48.2
	Probably true	NC	NA	442	44.2	428	42.8	371	37.1	374	37.4	379	37.9
	Probably false	NC	NA	69	6.9	66	6.6	72	7.2	68	6.8	68	6.8
	Definitely false	NC	NA	15	1.5	17	1.7	11	1.1	17	1.7	14	1.4
	Don’t know	NC	NA	110	11.0	82	8.2	91	9.1	73	7.3	58	5.8
**Total number of respondents and %**		**NC**	**NA**	**999**	**100**	**1,000**	**100**	**1,000**	**100**	**1,000**	**100**	**1,001**	**100**
Likelihood to ask your GP for antibiotics	Very likely	79	7.9	58	5.8*^,a^	60	6.0	70	7.0	90	9.0	91	9.1
	Quite likely	124	12.4	106	10.6*^,a^	134	13.4	117	11.7	118	11.8	129	12.9
	Quite unlikely	278	27.8	246	24.6*^,a^	246	24.6	219	21.9	233	23.3	235	23.5
	Very unlikely	448	44.8	531	53.1*^,a^	508	50.8	528	52.7	485	48.5	485	48.5
	Don’t know	70	7.0	59	5.9^*,a^	52	5.2	67	6.7	73	7.3	60	6.0
**Total number of respondents and %**		**999**	**100**	**1,000**	**100**	**1,000**	**100**	**1,001**	**100**	**999**	**100**	**1,000**	**100**
Parents													
I always take my GP's advice about whether my child needs antibiotics	Strongly agree	85	34.3	NC	NA	117	45.0	113	44.1	114	42.2	120	45.3
	Agree	128	51.6	NC	NA	116	44.6	111	43.4	122	45.2	112	42.3
	Disagree	21	8.5	NC	NA	19	7.3	19	7.4	23	8.5	21	7.9
	Strongly disagree	4	1.6	NC	NA	2	0.8	2	0.8	4	1.5	5	1.9
	Don’t know	10	4.0	NC	NA	6	2.3	11	4.3	7	2.6	7	2.6
**Total number of respondents and %**		**248**	**100**	**NC**	**NA**	**260**	**100**	**256**	**100**	**270**	**100**	**265**	**100**
Likelihood to ask your GP for antibiotics for others/your child	Very likely	56	15.0	51*^,c^	13.4	13	9.9	11	8.4	14	10.7	21	16.0
	Quite likely	89	23.9	63*^,c^	16.5	27	20.6	25	19.1	31	23.7	26	19.7
	Quite unlikely	96	25.7	102*^,c^	26.8	41	31.3	39	29.8	38	29.0	33	25.0
	Very unlikely	107	28.7	142*^,c^	37.3	45	34.4	45	34.4	37	28.2	43	32.6
	Don’t know	25	6.7	23*^,c^	6.0	5	3.8	11	8.4	11	8.4	9	6.8
**Total number of respondents and %**		**373**	**100**	**381**	**100**	**131**	**100**	**131**	**100**	**131**	**100**	**132**	**100**

GP: general practitioner; NA: not applicable; NC: data not collected at this time point; n: sample size.

^a^ *X^2^* and p values are included in Supplementary Material 7.

^b^ Flu: influenza.

^c^ *X^2^* and p values are included in Supplementary Material 8.

A significant difference (p < 0.05) is marked with an asterisk (*). Each year the questionnaire respondents were anonymous, so whether some people participated in the study at more than one collection point is unknown.

At the start of the campaign, more specific knowledge on antibiotics was poorer, including knowledge regarding appropriate antibiotic usage and the reduction of antibiotic effectiveness when they are taken inappropriately. There was a significant difference in responses to the statement ‘Antibiotics will stop working for you if taken for the wrong things’ following the campaign. The proportion of individuals answering true increased by 8.5% (net true: pre-2017 = 69.1%; post-2019 = 77.6%; *X^2^* = 5.753, p = 0.016). The proportion of individuals identifying that ‘Taking antibiotics when you don’t need them puts you and your family at risk of antibiotic resistant infections’ is true increased by 5.6% from post-2017 to post-2019 (net true: post-2017 = 80.5%; post-2019 = 86.1%; *X^2^* = 20.345, p < 0.001).

Overall, the proportion of individuals reporting some level of concern (5–7 on Likert scale) about antibiotic resistance for themselves increased by 11.2% from 39.1 (376/962) to 50.3% (656/1,304) (Z = −5.091, p < 0.001) pre- to post-campaign respectively (Table 6).

**Table 6:** Descriptive statistics showing level of concern about antimicrobial resistance assessed using a 7-point Likert scale, England 2017–2019

Concern about AMR	Pre 2017		Post 2017		Pre 2018		Post 2018		Pre 2019		Post 2019	
	Median	IQR	Median	IQR	Median	IQR	Median	IQR	Median	IQR	Median	IQR
For you*	4	3–5	4	3–5	4	3–5	4*	2–5	4	3–5	5	3–6
For children*	5	4–6	4*	3–5	5	4–6	4*	3–6	5	4–6	5	4–6

AMR: antimicrobial resistance; IQR: interquartile range.

A significant difference (p < 0.05) is marked with an asterisk (*). Z-scores and p values are included in Supplementary Material 9. Each year the questionnaire respondents were anonymous, so whether some people participated in the study at more than one collection point is unknown.

The campaign had some impact on parents’ awareness and understanding of AMR as the proportion of parents reporting some level of concern (5–7 on Likert scale) regarding AMR for children increased by 6.0% from 53.7 (495/922) to 59.7 (751/1,258) (Z = −3.616, p < 0.001) pre- to post-campaign respectively. However, the proportion of parents who agreed they would always take their doctor’s advice on whether their child needed antibiotics remained stable over the duration of the campaign.

### Change in reported behaviour

There was a significant 5.1% increase in the proportion of individuals reporting they were unlikely to ask for antibiotics following the first year of the campaign (*X^2^* = 6.067, p = 0.014) (Table 5). However, following the final year of the campaign, this returned to a similar level seen in the pre-2017 measure (pre-2017: 72.6% vs post-2019: 72.0%). There was also a significant increase of 9.6% in the proportion of parents reporting they were unlikely to ask for antibiotics for their child following the first year of the campaign (pre-2017: 54.4% (203/373); post-2017: 64.0% (244/381); *X^2^* = 7.645, p = 0.006). However, this reduced to 57.6% (76/132) following the final year of the campaign.

There were significant differences post-campaign in hot state actions (decision-making, which is influenced by the individuals emotional state which is likely to occur when they are unwell). A greater number of participants reported that they did not expect to receive antibiotics from their GP when ill (post-2018: 12.7% (83/655); post-2019: 16.8% (120/713); *X^2^* = 4.672, p = 0.031). Furthermore, a greater number of participants reported using non-urgent NHS services first, such as a walk-in centre, instead of seeing their GP when they fell ill (post-2018: 3% (19/655); post-2019: 5.3% (38/713), *X^2^* = 5.019, p = 0.025), as per the answer to Question 049 in Supplementary Material 2.

### General practitioners

Most GPs agreed that the campaign supported them to say no to patients asking for antibiotics and that the campaign will make patients less likely to ask for antibiotics. Furthermore, almost all GPs who recognised the campaign felt it helped raise awareness of AMR (Table 7).

**Table 7:** Attitudes towards the KAW campaign, prescribing attitudes and actions taken when inappropriately asked for antibiotics in GPs who were aware of the campaign, England 2017−2019

Statements and related opinions		2017 (n = 185)		2018 (n =178)		2019 (n = 206)	
		n	%	n	%	n	%
The advertising supports GPs to say no to patients asking for antibiotics when GPs think they are not needed	Strongly agree	48	25.9	40	22.6	49	23.8
	Agree	119	64.3	121	68.4	135	65.5
	Neither agree nor disagree	10	5.4	11	6.2	11	5.3
	Disagree	6	3.2	2	1.1	10	4.9
	Strongly disagree	2	1.1	3	1.7	1	0.5
**Total^a^**		**185**	**100**	**177^a^**	**100**	**206**	**100**
The advertising will make patients less likely to ask for antibiotics when you say they aren’t needed	Strongly agree	40	21.7	38	21.3	52	25.4
	Agree	108	58.7	106	59.6	106	51.7
	Neither agree nor disagree	18	9.8	21	11.8	23	11.2
	Disagree	11	6.0	10	5.6	18	8.8
	Strongly disagree	2	1.1	3	1.7	1	0.5
	Don’t know	5	2.7	0	0.0	5	2.4
**Total**		**184^a^**	**100**	**178**	**100**	**205^a^**	**100**
The advertising will help to raise awareness of the issue of AMR	Strongly agree	85	46.2	51	28.8	66	32.2
	Agree	95	51.6	122	68.9	128	62.4
	Neither agree nor disagree	1	0.5	0	0.0	7	3.4
	Disagree	2	1.1	2	1.1	3	1.5
	Strong disagree	1	0.5	2	1.1	1	0.5
**Total**		**184^a^**	**100**	**177^a^**	**100**	**205^a^**	**100**

AMR: antimicrobial resistance; GPs: general practitioners; n: sample size; NHS: National Health Service.

^a^ For some of the statements, summing up numbers in the corresponding columns results in slightly less or more counts than the total displayed in the column heading. These discrepancies result from weighting to allow comparisons between independent samples.

Each year the questionnaire respondents were anonymous, so whether some GPs participated in the study at more than one collection point is unknown.

In addition to this, following the campaign in 2017, GPs who were aware of the campaign were significantly more confident to say no to most patients when they requested antibiotics (98.9% (182/184) vs 92.4% (97/105), *X^2^* = 4.000, p = 0.045), however, this difference was no longer evident following the final year of the campaign. In 2018, a higher proportion of GPs who had seen the campaign explained the reasons why antibiotics are inappropriate when they were asked to prescribe antibiotics inappropriately (90.4% (161/178) vs 75.2% (82/109), *X^2^* = 9.217, p = 0.002), although this difference was also not evident post 2019 (Table 8).

**Table 8:** Prescribing attitudes and actions taken by general practitioners when inappropriately asked for antibiotics, stratified by those who were exposed or not to the KAW-campaign, England, 2017–2019

Statements and related opinions		2017				2018				2019			
		Aware of campaign (n = 184)		Unaware of campaign (n = 105)		Aware of campaign (n = 178)		Unaware of campaign (n = 109)		Aware of campaign (n = 206)		Unaware of campaign (n = 137)	
		n	%	n	%	n	%	n	%	n	%	n	%
I am confident I can say no to most patients asking for antibiotics when I don’t think they are needed	Strongly agree	77	41.8	37*	35.8	61	34.1	42	38.5	77	37.3	57	41.6
	Agree	105	57.0	60*	57.1	113	63.9	64	58.7	115	56.0	73	53.3
	Disagree	2	1.2	2*	1.7	3	1.9	0	0.0	7	3.2	4	2.9
	Strongly disagree	0	0	3*	2.6	1	0.6	0	0.0	6	3.0	3	2.2
	Don’t know	0	0	3*	2.8	0	0.0	3	2.8	1	0.6	0	0.0
**Total**		**184**	**100**	**105**	**100**	**178**	**100**	**109**	**100**	**206**	**100**	**137**	**100**
Explained the reasons why antibiotics are inappropriate	Yes	175	95.1	91	86.7	161	90.4	82*	75.2	194	94.4	123	89.8
	No	9	4.9	10	9.5	17	9.6	24*	22.0	11	5.6	11	8.0
	Missing	0	0.0	4	3.8	0	0.0	3	2.8	0	0.0	3	2.2
**Total**		**184**	**100**	**105**	**100**	**178**	**100**	**109**	**100**	**205^a^**	**100**	**137**	**100**

AMR: antimicrobial resistance; KAW: Keep Antibiotics Working; n: sample size; NHS: National Health Service.

^a^ The total in this cells is less than the total displayed in the column heading because of weighting to allow comparisons between independent samples.

Significant difference (p < 0.05) are outlined by an asterisk (*); *X^2^* and p-values are included in Supplementary Material 10. Each year the questionnaire respondents were anonymous, so whether some general practitioners participated in the study at more than one collection point is unknown.

## Discussion

Our main findings are firstly, that campaign recognition increased following the 3-year campaign with slight differences seen in levels of recognition between the general public and within key subgroups. Adults over 50 years had significantly lower levels of unprompted recognition following the 2018 and 2019 campaign years, although there was no difference in their level of prompted recognition. Reasons for this are unclear but may be due to employed methods of communication with social media used more extensively to disseminate information in 2018 and 2019, or differing levels of interest in the campaign affecting participant recall. The level of prompted recognition for the present campaign was high with TV being the most common source of recognition, supporting the use of mass media campaigns to disseminate information to the public. This level of recognition was higher than for previous national antibiotic awareness campaigns “The English public antibiotic campaigns” [4], and similar to that of the successful “Change4Life” social marketing campaign [20]. Interestingly, unprompted campaign recognition in the pre-2017 measure was 13% when it would be expected to be 0%. This may be due to some individuals recognising campaign material from within the region that the pilot campaign was conducted, or individuals may have seen other promotional material related to AMR from other campaigns which were running concurrently, such as Antibiotic Guardian [9].

Secondly, aspects of participant knowledge, awareness and understanding of AMR increased following the campaign. Perceived knowledge of both antibiotic and antimicrobial resistance increased post-campaign. Perceived knowledge of AMR was lower before the campaign but increased to a greater extent. The differences in knowledge are likely due to an unfamiliarity with the term ‘antimicrobial resistance’ as it is typically more commonly used by subject experts, with public campaigns predominately focusing on the role of antibiotics and bacterial resistance to this class of drug [4] as this is where the burden of resistant infections falls [21]. In addition to improvements in perceived knowledge, understanding of more specific antibiotic topics also increased with an additional 8.5 and 5.6% of participants correctly identifying that “Antibiotics will stop working if taken for the wrong things” and “Taking antibiotics when you don’t need them puts you and your family at risk of antibiotic resistant infections” are true. The level of concern about AMR for self and for children also saw year-on-year increases, showing improvements in awareness among the public of the personal risk of AMR following the campaign. This increase may be important due to the influence that concern can have over future behaviour by encouraging individuals to seek further information on a topic [22].

Previous research has shown that messaging related to a specific disease, such as respiratory tract infections (RTIs), may be more effective at reducing inappropriate antibiotic usage than more generic messaging [23]. Despite the ineffectiveness of antibiotics at treating viral infections, such as RTIs, being one of the KAW campaigns key messages, a decline in knowledge on this topic was seen following the campaign. This suggests messaging related to diseases which are treatable with antibiotics may not have been clear.

A positive effect on reported behavioural intentions was seen following the campaign with respondents reporting being less likely to expect an antibiotic prescription and the proportion of participants reporting that they would go to a pharmacy or use 111, a non-emergency number for urgent healthcare need that is not a life-threatening situation, because of seeing the campaign increased. Reported likelihood to request antibiotics was also affected with a 5.1 and 9.6% increase in the proportion of individuals reporting they were unlikely to request antibiotics from their GP for themselves or their child following the first year of the campaign, respectively. However, the significant differences which occurred following the 2017 wave of the campaign returned to baseline following the final year of the campaign. This suggests that a longer campaign duration may not result in greater improvements in reported knowledge, attitudes, and behaviours. It is difficult to determine the cause of the lack of further change in knowledge and attitudes in 2018 and 2019. However, the 50% reduction in spend and the year-on-year reduction in campaign video views from 10.3 million in 2017 to 5.6 million in 2019 may have contributed to this. Furthermore, some campaign fatigue was present by the end of the third year of advertising which may have also influenced attention paid to campaign messages [23]. Future content and messages could be refreshed to address this; previous research has also shown that allowing your target audience to influence campaign content may increase engagement [24,25].

Finally, in the first 2 years of the campaign, KAW supported change among GPs with GPs who were aware of the campaign reporting greater confidence to say no to most patients asking for antibiotics. GPs who were aware of the campaign were also more likely to explain that prescription of antibiotics for viral infections was inappropriate when asked for antibiotics by a patient. Almost all GPs felt the campaign was important and helped to raise awareness of AMR. Supporting GPs and providing resources that can be used to facilitate healthcare professional and patient interactions in combination with messaging focused on the public has been shown to result in significant reductions in antibiotic usage [26]. However, despite the positive reception from GPs, 44% of GPs surveyed in 2019 reported they are still frequently asked to prescribe antibiotics when they are not needed and they still feel pressure to do so [11].

Overall, the campaign evaluation showed the key aims of the campaign were met with several significant changes in knowledge, attitudes, concern about AMR, and intentions to alter behaviour which would improve appropriate antibiotic usage and reduce pressure on GPs to prescribe unnecessary antibiotics.

This study presents a comprehensive evaluation of a national AMR awareness campaign which are often not published. Nevertheless, the study does have some limitations. Firstly, the differing samples interviewed before and after each campaign year introduces individual variability; weighting of the samples attempted to reduce this variability. The use of self-report measures introduces the potential for social desirability bias in responses to attitude and behaviour-based questions. The lack of baseline measure for some variables also makes it difficult to determine the level of effect the KAW campaign had on all aspects of participant knowledge, attitudes, and behaviour.

Furthermore, a sample size calculation was not completed. However, the sample sizes were deemed to provide a sufficiently large base size to allow robust analysis both of the overall sample and of key subgroups, while considering cost.

Finally, the observational nature of the study and the lack of measurable behaviour change resulting from viewing the campaign messages means causation is difficult to determine. Several measures of changes in behaviour have been used in previous campaign evaluations, including GP attendance for colds, uptake of influenza vaccination, and antimicrobial usage [27]. One such proxy measure of change which can be used is prescribing rates. Following an analysis of national antibiotic prescribing data, antibiotic prescribing reduced by 15.1% from 18.8 Daily Defined Dose per 1,000 inhabitants per day (DID) to 15.9 DID from 2017 to 2021 [21]. Campaigns of a similar scale, although run for a longer period of time, have produced similar reductions in antibiotic prescribing. A campaign run in Belgium used mass media to improve public understanding of self-limiting infections, the need to use antibiotics appropriately and the consequences of AMR. It also aimed to facilitate discussions between patients, clinicians, and pharmacists. When evaluated in 2018, the campaign was recognised among 44.6% of participants and a 12.8% reduction in antibiotic prescribing was observed since the campaign inception in 2000 [26]. A 12.6% reduction in antibiotic prescribing 14 years post-campaign was also seen following a similar national awareness campaign run in France [28]. The apparent success of these campaigns provides support for the use of social-marketing campaigns to have a positive impact on antimicrobial usage which is likely to have a subsequent effect on AMR.

Changes in healthcare seeking behaviour have occurred as a result of COVID-19, which, in combination with pandemic restrictions, have resulted in a reduction in antibiotic-resistant bloodstream infections and antibiotic prescribing [29]. Therefore, due to the impact of the COVID-19 pandemic on healthcare, among other confounding variables, the direct effect of KAW on antibiotic prescribing cannot be determined. Further research is needed to ascertain effective ways of disseminating information and methods to determine a measurable behaviour change resulting from AMR campaigns and the economic assessment of public campaigns on AMR [30,31].

Changes in the public’s understanding of infection prevention and hand hygiene following the COVID-19 pandemic, as well as its positive impact on attitudes towards vaccination presents an opportunity for AMR messaging and interventions [32,33]. Future campaigns should use the momentum generated by COVID-19 messaging to raise awareness of the global risk of AMR.

### Conclusions

This paper highlights the strength of a multimedia, integrated social marketing and communications campaign in reaching its target audience, increasing awareness, and supporting GPs, and demonstrates that KAW was an important component in tackling AMR. The COVID-19 pandemic has changed the health landscape since the KAW campaign was launched, and further research should be undertaken to understand current attitudes towards and use of antibiotics, and to determine measurable behaviour outcomes to inform and allow the success of future campaigns to be determined with more certainty.

## Supplementary Data

1265000 Supplement
