# Supplementary material for: Assessing the impact of a national social marketing campaign for antimicrobial resistance on public awareness, attitudes, and behaviour, and as a supportive tool for healthcare professionals, England, 2017 to 2019
Source: Euro Surveill. 2023 Nov 23;28(47):2300100. doi: 10.2807/1560-7917.ES.2023.28.47.2300100 (PMC10668255; doi:10.2807/1560-7917.ES.2023.28.47.2300100)
Supplement: Supplement [file 23-00100_ASHIRU_Supplement.pdf]

This supplementary material is hosted by *Eurosurveillance* as supporting information alongside the article Assessing the impact of a national social marketing campaign for antimicrobial resistance on public awareness, attitudes, and behaviour, and as a supportive tool for healthcare professionals, England, 2017 to 2019, on behalf of the authors, who remain responsible for the accuracy and appropriateness of the content. The same standards for ethics, copyright, attributions and permissions as for the article apply. Supplements are not edited by *Eurosurveillance* and the journal is not responsible for the maintenance of any links or email addresses provided therein.

KANTAR

Survey:
Version: 1
Global Practice: ...

© Kantar 4-11-2019

Supplementary Material 1. Keep Antibiotics Working social media video link

[Antibiotic resistance advert - keep antibiotics working and take your doctor's advice - YouTube]

Supplementary Image 1. Keep Antibiotics Working checklist poster

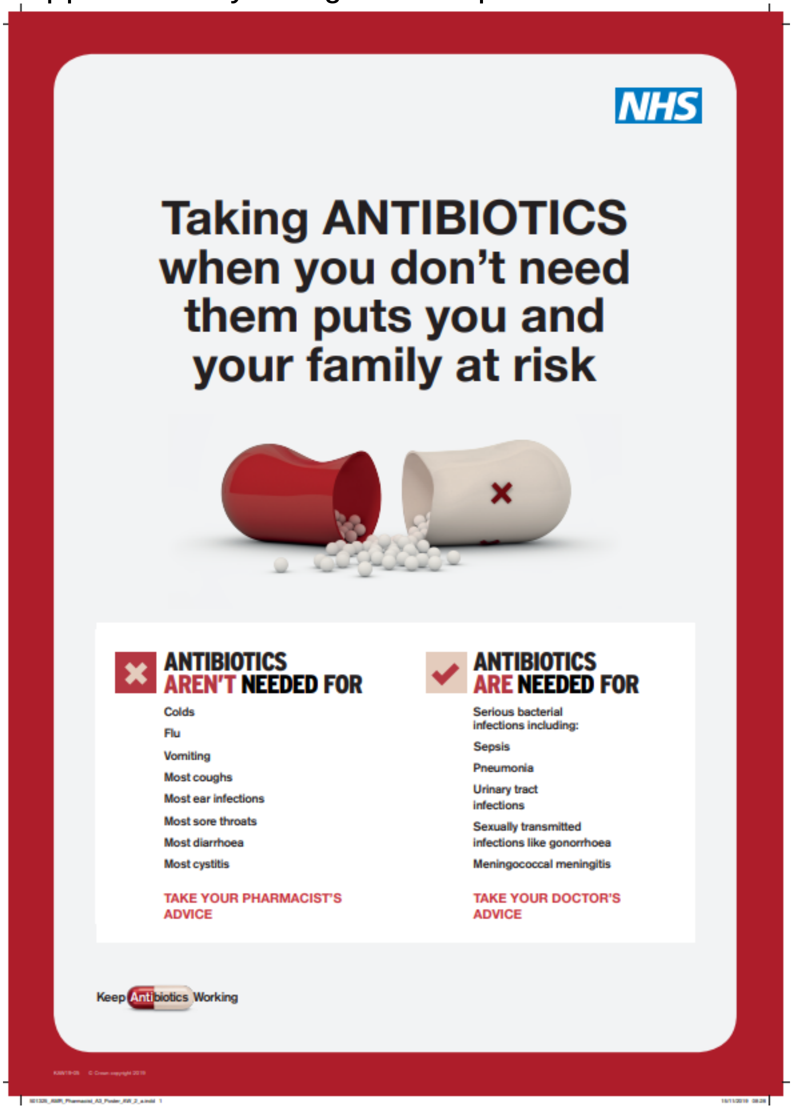

Supplementary Image 2.

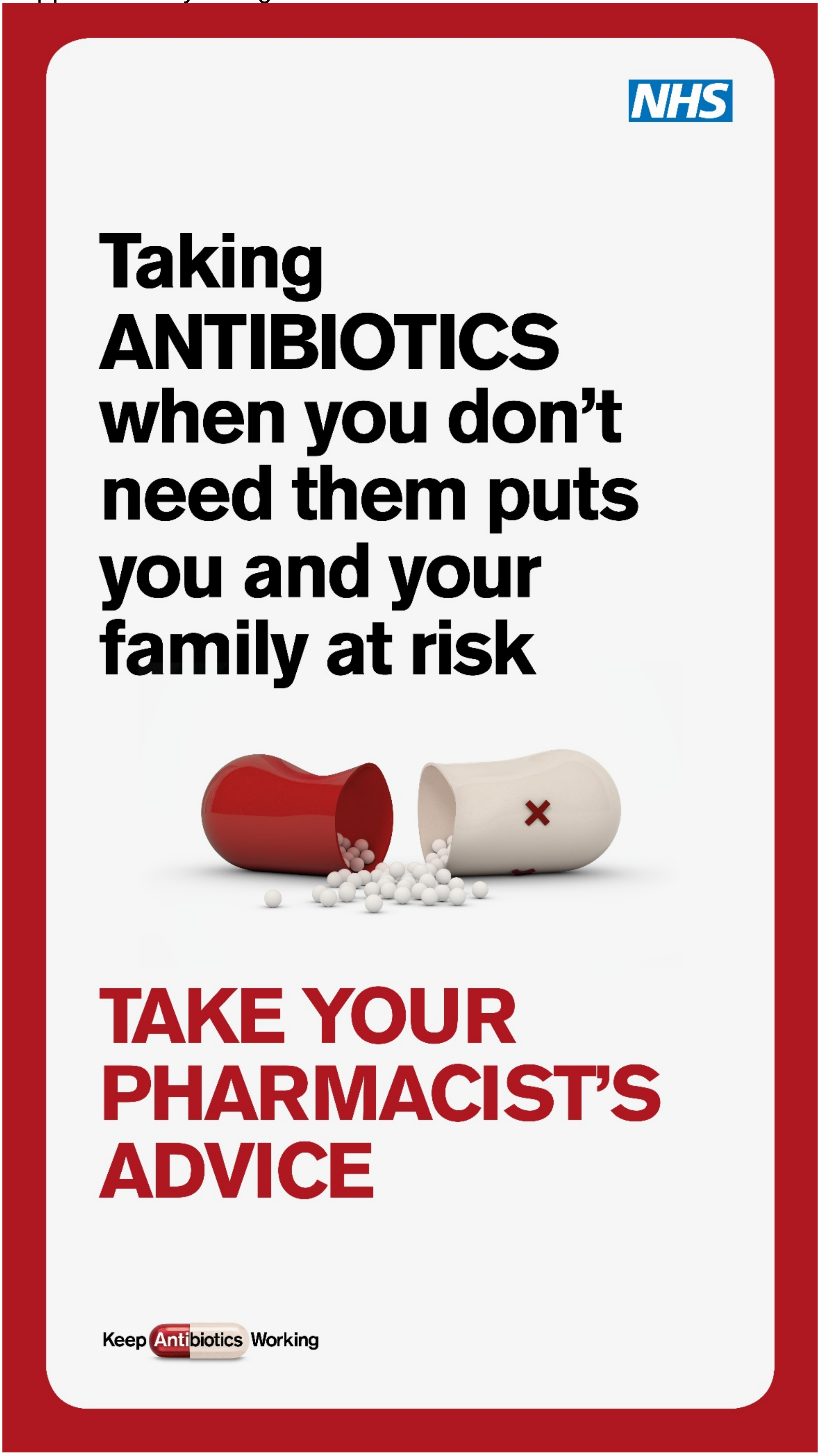

Supplementary Material 2. CAWI Questionnaire

Name of survey
AMR Post-stage CAWI questionnaire 2019 v1

Client name
[redacted]

Author(s)
[redacted]

This questionnaire was written according to Kantar quality procedures

checked by

[redacted]

Repeating study (if this survey has been previously conducted)	Yes – AMR pilot – 260138556<br>AMR national rollout – 260142800<br>AMR 2018 - 40303453
Name of survey	AMR gen pop post-stage CAWI questionnaire
Language	English (United Kingdom)
Survey length (minutes)	15
Version	2
Author(s)	[redacted]
Contact	
Panel	LSR
Sample size	Gross: 1,350 per wave<br>Net:
Sample description	General public in England (1,000)<br>Boost in Granada (200)<br>Boost of mothers of 0-16s in England (150)
Quota	
If several countries: indicate the countries	
If several targets	
Check-in site	[redacted]
Comments	

Q001 - Q001: Intro — Text

Not back

Welcome to the survey!

This survey will take up to 15 minutes. Please click on the button below to begin the survey.

Q002 - Q017_Copy_1: Intro 2 — Text

Not back

Thank you for agreeing to take part in this survey.

Please do not use the refresh, back or forward buttons on your browser during the survey, and do not use your keyboard except when you are asked to type in a response. Instead, please just use your mouse or any buttons within the survey itself.

If possible, please complete the survey in one sitting, since you will not be able to access it at a later time.

Please maximise this window before you continue.

Q003 - Q042: AGE — Numeric

Not back | Min = 20 | Max = 100

How old are you?

Scripter notes: Add an answer option for 'Prefer not to say'

Screen out if age < 18

Q004 - Q043: Age range — Single coded

Not back

Which of the following age groups do you fall into?

Normal

1	Under 18<br>↳ GO TO SCREEN OUT
2	18-19
3	20-25
4	26-35
5	36-45
6	46-50
7	51-60
8	61-70
9	71+
10	Prefer not to say

Scripter notes: ASK this question IF at Q42 = 'Prefer not to say'

© Kantar 4-11-2019

Q005 - Q044: Sex — Single coded

Not back

Are you...?

Normal

1 Male
2 Female
3 Prefer not to say

Q006 - Q045: Region — Single coded

Not back

Whereabouts in the country do you live?

Normal

1 North East
2 North West
3 Yorkshire and the Humber
4 East Midlands
5 West Midlands
6 East of England
7 South East excluding London
8 London
9 South West
11 Not in England
– GO TO SCREEN OUT
10 Prefer not to say
↳ GO TO SCREEN OUT

Scripter notes: Granada variable
Q006 = 2

Q007 - Q29: Carer status — Multi coded

Do you currently have caring responsibilities for any of the following people?

By caring responsibilities we mean being responsible for decisions for friends and family (not as part of your work) that affect their education, health, or well-being.

Please select all that apply.

Normal

1 Child(ren) aged 4 or younger
2 Child(ren) aged 5-11
3 Child(ren) aged 12-16
4 Elderly parents or other relatives
5 Spouse
6 Other **Position fixed*
98 None of the above **Position fixed *Exclusive*
99 Don't know **Position fixed *Exclusive*

Scripter notes:
Mothers of 0-16s – variable:
Q005 = 2
AND
Q007 = 1/2/3

Q008 - Q046: Social Grade | Single coded

Not back

Which of the following groups does the Chief Income Earner in your household belong to….

Normal

1 Semi or unskilled manual worker
2 Skilled manual worker
3 Supervisory or clerical/ Junior managerial/ Professional/ administrator
4 Intermediate managerial/ Professional/ Administrative
5 Higher managerial/ Professional/Administrative
6 Student
7 Retired and living on state pension only
8 Unemployed (for over 6 months) or not working due to long term sickness
9 Housewife / Househusband / Homemaker
10 Prefer not to say
↳ GO TO SCREEN OUT

Scripter notes: Scripter notes: Text: "Who is the Chief Income Earner?"
Show hover over: "The person in the household with the largest income is the Chief Income Earner, however this income is obtained."
For responses, SHOW TEXT IN BRACKETS WHEN CODE IS HOVERED OVER
1.Semi or unskilled manual worker (e.g. manual jobs that require no special training or qualifications; manual workers, apprentices to be skilled trades, caretaker, cleaner, nursery school assistant, park keeper, non-HGV driver, shop assistant etc.)
2.Skilled manual worker (e.g. Skilled Bricklayer, Carpenter, Plumber, Painter, Bus/Ambulance Driver, HGV driver, Unqualified assistant teacher, AA patrolman, pub/bar worker, etc.)
3.Supervisory or clerical/ Junior managerial/ Professional/ administrator (e.g. Office worker, Foreman with 25+ employees, sales person, etc.)
4.Intermediate managerial/ Professional/ Administrative (e.g. Newly qualified (under 3 years) doctor, Solicitor, Board director small organisation, middle manager in large organisation, principle officer in civil Service/local government etc.)
5.Higher managerial/ Professional/Administrative (e.g. Established doctor, Solicitor, Board Director in large organisation (200+ employees, top level civil servant/public service employee, headteacher, etc.)
7.Retired and living on state pension only (If the Chief Income Earner is retired and has an occupational pension, please select according to their previous occupation)
8.Unemployed (for over 6 months) or not working due to long term sickness (If the Chief Income Earner is not in paid employment and has been out of work for less than 6 months, please select according to previous occupation)

Q009 - Q027: Health care professionals — Single coded

Not back

Do you work in any of the following professions?

Random

1 Nurse
↳ GO TO SCREEN OUT
2 Doctor
↳ GO TO SCREEN OUT
3 Pharmacist
↳ GO TO SCREEN OUT
8 Dentist
↳ GO TO SCREEN OUT
4 Teacher
5 Engineer
6 Police officer
7 Solicitor
98 None of the above **Position fixed *Exclusive*

Q010 - Q039: Frequency of antibiotics use — Matrix

Not back | Number of rows: 6 | Number of columns: 5

In the last year, approximately how many times have you done each of the following?

Random

Rendered as Dynamic Grid

	Zero	1-3	4-10	11+	Don't know
Visited the GP	❍	❍	❍	❍	❍
Taken antibiotics prescribed by a GP at your usual practice	❍	❍	❍	❍	❍
Taken antibiotics prescribed by a dentist	❍	❍	❍	❍	❍
Asked the pharmacist for advice on how to treat an illness	❍	❍	❍	❍	❍
Taken antibiotics from somewhere else (eg online or from friends/family)	❍	❍	❍	❍	❍
Taken antibiotics I had left over from something else	❍	❍	❍	❍	❍

Ask only if **Q007 - Q29**,1,4,5,2,3

Q011 - Q045_Copy_1: Frequency of antibiotics use for dependents | **Matrix**

Not back | Number of rows: 6 | Number of columns: 5

In the last year, approximately how many times have you done the following for somebody else? By 'somebody else' we mean any children you might have or any other people who you look after, but not as part of your work.

Random

Rendered as Dynamic Grid

	Zero	1-3	4-10	11+	Don't know
Visited the GP	❍	❍	❍	❍	❍
Given antibiotics prescribed by a GP at your usual practice	❍	❍	❍	❍	❍
Given antibiotics prescribed by a dentist	❍	❍	❍	❍	❍
Asked the pharmacist for advice on how to treat an illness	❍	❍	❍	❍	❍
Given antibiotics from somewhere else (eg online or from friends/family)	❍	❍	❍	❍	❍
Gave antibiotics I had left over from something else	❍	❍	❍	❍	❍

Q012 - Q034: Knowledge about what ABs can be used to treat | **Multi coded**

Not back | Min = 1

To the best of your knowledge, which of the following conditions do not usually need to be treated by antibiotics?

Random

1	Common cold
2	Flu
3	Cough or bronchitis
4	Sexually Transmitted Infections (STIs) like gonorrhoea
5	Food poisoning (e.g. E-coli)
6	Meningococcal meningitis
7	Vomiting
8	Cystitis
9	Urinary Tract Infections (UTIs)
10	Ear infections/earache
13	Bacterial meningitis
14	Diarrhoea
15	Pneumonia
16	Sepsis
22	Sinusitis
17	Sore throat
18	Fungal infections such as athlete's foot, thrush or ringworm
19	Allergic reactions (such as bee stings)
20	Hay fever
21	Asthma
22	Toothache
11	None of the above
12	Don't know

Q050 - UnwellFirst: Unwell first action | **Single coded**

Imagine the next time you feel unwell and are unsure if you need antibiotics or something else which, if any, of the following would you do **first**?

Random

1	Speak to a friend or family member for advice
2	Look for information online
3	Buy some non-prescription medicine without seeking medical advice
4	Care for myself at home
5	Visit a chemist/pharmacy for advice about my condition
6	Make an appointment to see my/a GP
7	Phone NHS 111
8	Go to A&E
9	Phone 999/Ambulance
10	Order antibiotics online
11	Take antibiotics I already have / prescribed for someone else in my household
12	Do something else (please specify)
13	Do nothing unless the condition showed any signs of getting worse **Position fixed *Exclusive*
14	Don't know **Position fixed *Exclusive*

ASK IF Q051 – UnwellFirst = 1-12

Q051 - UnwellOther: Unwell other actions | **Multi coded**

And what else would you do, the next time you feel unwell and are unsure if you need antibiotics or something else?

Random

1	❍	Speak to a friend or family member for advice
2	❍	Look for information online
3	❍	Buy some non-prescription medicine without seeking medical advice
4	❍	Care for myself at home
5	❍	Visit a chemist/pharmacy for advice about my condition
6	❍	Make an appointment to see my/a GP
7	❍	Phone NHS 111
8	❍	Go to A&E
9	❍	Phone 999/Ambulance
10	❍	Order antibiotics online
11	❍	Take antibiotics I already have / prescribed for someone else in my household
12	❍	Do something else (please specify)
13	❍	Do nothing unless the condition showed any signs of getting worse **Position fixed *Exclusive*
14	❍	Don't know **Position fixed *Exclusive*

Scripter notes: Do not show code selected at Unwell first action
Codes display in same order as Unwell first action
Group codes 8-9 and 10-11
Fix 13 and 14 at end

Q014 - Q041: Likelihood to ask for ABs	Single coded

Not back

Imagine you feel unwell and make an appointment to see your GP. At the time of the appointment you are sure that you need antibiotics to treat the illness, but the GP says they do not think you need them.

How likely or unlikely would you be to ask the GP to prescribe you antibiotics anyway?

Normal

1	Very likely
2	Quite likely
3	Quite unlikely
4	Very unlikely
99	Don't know **Position fixed *Exclusive*

Scripter notes: FLIP CODES 1-4 FOR 50% RESPONDENTS

ASK IF Q014 – Q041: Likelihood to ask for ABs = 1,2

Q052 – Likelihood_likely: Likelihood follow up - likely	Multi coded

Not back

You said you would be [very/quite] likely to ask the GP to prescribe you antibiotics anyway. Why would you do this?

Random

1	I would worry it might be more serious
2	It’s better to get antibiotics just in case
3	I know when I need antibiotics/I know I need antibiotics by how I’m feeling
4	I wouldn’t want to waste my time by having to come back again
5	I wouldn’t want to waste the GP’s time by having to come back again
6	I don’t trust GPs’ advice/GPs aren’t always right
7	GPs don’t have time to assess you properly
8	Other (specify)
99	Don't know **Position fixed *Exclusive*

Scripter notes:

ASK IF Q014 – Q041: Likelihood to ask for ABs = 3,4

Q053 – Likelihood_unlikely: Likelihood follow up - unlikely	Multi coded

Not back

You said you would be [very/quite] unlikely to ask the GP to prescribe you antibiotics anyway. Why is this?

Random

1	I trust the GP to make the right decision
2	I would feel relieved that I don't need antibiotics
3	Taking antibiotics gets in the way of my day to day life
5	I am worried / that antibiotics will stop working for me if I take them when I don't need to
7	Other (specify)
99	Don't know **Position fixed *Exclusive*

Scripter notes:

Ask only if **Q005=2** AND **Q007 - Q29**,1,2,3

Q015 - Q033_Copy_1: Copy of Q024 - Q041: Likelihood to ask for ABs for other people	Single coded

Not back

Now imagine your child is unwell and you make an appointment to see your GP. At the time of the appointment you are sure that your child antibiotics to treat the illness, but the GP says they do not think they need them.

How likely or unlikely would you be to ask the GP to prescribe your child antibiotics anyway?

Normal

1	Very likely
2	Quite likely
3	Quite unlikely
4	Very unlikely
99	Don't know **Position fixed *Exclusive*

Scripter notes: FLIP CODES 1-4 FOR 50% RESPONDENTS

ASK IF Q015 – Q033: = 1,2

Q054 – Likelihood_other_people_likely: Likelihood other people follow up - likely	Multi coded

Not back

You said you would be [very/quite] likely to ask the GP to prescribe antibiotics for your child anyway. Why would you do this?

Random

1	I would worry it might be more serious
2	It's better to get antibiotics just in case
3	I know when my child needs antibiotics/I know my child need antibiotics by how they are feeling
4	I wouldn't want to waste my time by having to come back again
5	I wouldn't want to waste the GP's time by having to come back again
6	I don't trust GPs' advice/GPs aren't always right
7	GPs don't have time to assess my child properly
8	Other (specify)
99	Don't know **Position fixed *Exclusive*

Scripter notes:

ASK IF Q015 – Q033: = 3,4

**Q055 - Likelihood_other_people_unlikely: Likelihood other people follow up - unlikely**	**Multi coded**

Not back

You said you would be [very/quite] unlikely to ask the GP to prescribe antibiotics for your child anyway. Why is this?

Random

1	I trust the GP to make the right decision
2	I would feel relieved that my child doesn’t need antibiotics
3	Taking antibiotics gets in the way of my/my child’s day to day life
4	I wouldn’t want to pay for an antibiotic prescription
5	I am worried that antibiotics will stop working for my child if they take them when they don’t need to
7	Other (specify)
99	Don't know **Position fixed *Exclusive*

Scripter notes:

**Q018 - Q034_Copy_1: Social norms re pressure**	**Single coded**

Not back

Thinking now about people you know - which of the following do you think they would do if their GP said antibiotics weren’t needed?

Normal

1	Insist that the GP prescribe them antibiotics anyway
2	Ask the GP for antibiotics but not insist on a prescription
3	Accept the GP’s view that antibiotics aren’t needed and not ask for antibiotics
4	None of the above

Scripter notes: FLIP CODES 1-3 FOR 50% RESPONDENTS

Q019 - Q035: Attitudes/knowledge about antibiotics Matrix

Not back | Number of rows: 16 | Number of columns: 5

Here are some things people have said about antibiotics. Please indicate to what extent you agree or disagree with each.

Random

Rendered as Dynamic Grid

	Strongly agree	Tend to agree	Tend to disagree	Strongly disagree	Don't know
It's ok to take antibiotics originally prescribed for someone else	❍	❍	❍	❍	❍
I always take my doctor's advice about whether I need antibiotics or not	❍	❍	❍	❍	❍
I **always** expect the doctor to give me antibiotics if I ask for them	❍	❍	❍	❍	❍
**Most of the time**, I expect the doctor to give me antibiotics if I ask for them	❍	❍	❍	❍	❍
I always take my doctor's advice about whether my child needs antibiotics or not (PARENTS ONLY)	❍	❍	❍	❍	❍
I expect the doctor to give me antibiotics for my child if I ask for them (PARENTS ONLY)	❍	❍	❍	❍	❍
Antibiotics always speed up my recovery, no matter what the illness is	❍	❍	❍	❍	❍
If there is any doubt about whether an illness needs to be treated with antibiotics it's better to take them just in case	❍	❍	❍	❍	❍
I trust the GP to make the right decision about whether I need antibiotics or not	❍	❍	❍	❍	❍
I would be relieved/happy if the GP said I didn't need antibiotics	❍	❍	❍	❍	❍
I would be relieved/happy if the GP said my child didn't need antibiotics (PARENTS ONLY)	❍	❍	❍	❍	❍
Antibiotics don't work for everything	❍	❍	❍	❍	❍

Scripter notes: Please keep don't know separate and show it as a radio button
Ask statements 6 & 7 if Q007 = 1/2/3

Q021 - Q037:	Matrix

Not back | Number of rows: 7 | Number of columns: 5

And looking at each of the following statements - to the best of your understanding, which do you think are true and which are false?

Random

Rendered as Dynamic Grid

	Definitely true	Probably true	Probably false	Definitely false	Don't know
There is nothing individuals can do to prevent antibiotics becoming less effective at treating diseases	❍	❍	❍	❍	❍
Antibiotics becoming less effective at treating diseases is not a problem in my area	❍	❍	❍	❍	❍
New antibiotics will always be developed to replace those that don't work any more	❍	❍	❍	❍	❍
Bacteria that are resistant to antibiotics spread easily from person to person	❍	❍	❍	❍	❍
Antibiotics will stop working for you if taken for the wrong things	❍	❍	❍	❍	❍
Taking antibiotics when you don't need them puts you and your family at risk of antibiotic resistant infections	❍	❍	❍	❍	❍
If children take antibiotics, the next time they get an infection it is more likely to be resistant (more so than adults) (PARENTS ONLY)	❍	❍	❍	❍	❍
You should not buy antibiotics online without a valid prescription	❍	❍	❍	❍	❍
Tackling antibiotic resistance should be one of the most important priorities for the NHS	❍	❍	❍	❍	❍
Antibiotic resistance occurs when new strains of bacteria develop and stop antibiotics from working	❍	❍	❍	❍	❍
Me using antibiotics won't impact overall antibiotic resistance	❍	❍	❍	❍	❍
There's nothing I can do to prevent antibiotics becoming less effective at treating diseases	❍	❍	❍	❍	❍

Scripter notes: Please pair statements 1 & 2 and fix at top of list, randomise within pair

Q022 - Q030: Familiarity - comparators — Matrix

Not back | Number of rows: 4 | Number of columns: 5

Before today, how much would you say that you know, if anything, about each of the following terms?

Normal

Rendered as Dynamic Grid

	Know a lot	Know something	Heard of but don't know anything about	Never heard of	Don't know
Antimicrobial resistance	❍	❍	❍	❍	❍
Antibiotic resistance	❍	❍	❍	❍	❍
AMR	❍	❍	❍	❍	❍
Superbugs	❍	❍	❍	❍	❍

Scripter notes: SCRIPT DK AS RADIO BUTTON
FLIP COLUMNS 1-4 FOR 50% RESPONDENTS

Q023 - Q033: Level of concern among those unaware of AMR — Matrix

Not back | Number of rows: 6 | Number of columns: 8

How concerned, if at all, are you about antibiotic resistance for each of the following?

Please answer on a scale of one to seven, where 1 is not at all concerned and 7 is very concerned.

Normal

Rendered as Dynamic Grid

	1 - Not at all concerned	2	3	4	5	6	7 - Very concerned	Don't know
You personally	❍	❍	❍	❍	❍	❍	❍	❍
Your family	❍	❍	❍	❍	❍	❍	❍	❍
Young children	❍	❍	❍	❍	❍	❍	❍	❍

Q024 - Q016_Copy_1: AMR publicity awareness — Single coded

Not back

Antibiotic resistance (sometimes described as antimicrobial resistance or AMR) is when bacterial infections become resistant to antibiotics, meaning even simple illnesses can become untreatable with those antibiotics.

Have you seen or heard anything about antibiotics or antibiotic resistance in the last six weeks or so?

Normal

1 Yes
2 No
99 Don't know **Position fixed *Exclusive*

Ask only if **Q024 - Q016_Copy_1**,1

Q025 - Q019_Copy_1: Prompted sources of awareness | **Multi coded**

Not back | Min = 1

Where did you see or hear something relating to antibiotics or antibiotic resistance?

Please select all that apply

Normal

1	TV programme
2	TV advertising
3	Newspaper article
4	Newspaper advertising
5	Magazine article
6	Magazine advertising
7	Radio
8	On a poster
9	Leaflet
10	Website content
11	On a blog
27	Video ad online
12	Other advertising on the internet
13	Facebook
14	Twitter
28	Instagram
15	Other social media
16	A friend/relative told me about it
17	My GP told me about it
18	A pharmacist/chemist told me about it
19	Another healthcare professional (e.g. dentist/nurse) told me about it
20	At / through work
21	At a doctor’s surgery/clinic
22	At a chemist/pharmacist
23	At a supermarket in store event
24	At another local event
25	Other (specify) **Open*
26	Don't know

Ask only if **Q024 - Q016_Copy_1**,1

Q026 - Q015_Copy_1: Description of what saw or heard | **Open**

Not back

And what was the focus of what you saw or heard about antibiotics or antibiotic resistance?

Please answer as fully as possibly in the box below.

Q034 - NEW1: **Text**

Not back

Now you will be shown some ads. Please watch carefully as we will be asking some questions on your views on the adverts, whether you have seen them before or not.

Q035 - NEW2: **Multi coded**

Not back

Have you seen this ad in the last couple of months?

Show movies: Play AMR video ad

Normal

1	Remember seeing this ad – in the last couple of months
4	Remember seeing this ad – longer ago
2	Do not remember seeing this ad
3	Don’t know

Q036 - NEW3: **Open**

Not back

What do you think was the main message of the ad you have just seen?

99 don't know **Position fixed *Exclusive*

Q037 - NEW4: **Multi coded**

Not back

We will now show you a radio ad. Please press play to listen. Have you heard this ad in the last couple of months?

Normal

1	Remember hearing this ad – in the last couple of months
4	Remember hearing this ad – longer ago
2	Do not remember hearing this ad
3	Don’t know

Q038 - NEW5: **Multi coded**

Not back | Min = 1

Have you seen these ads, or anything similar, in the last couple of months?

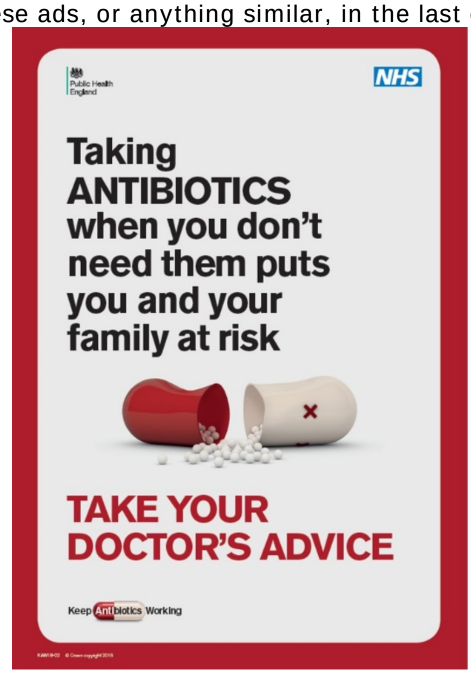

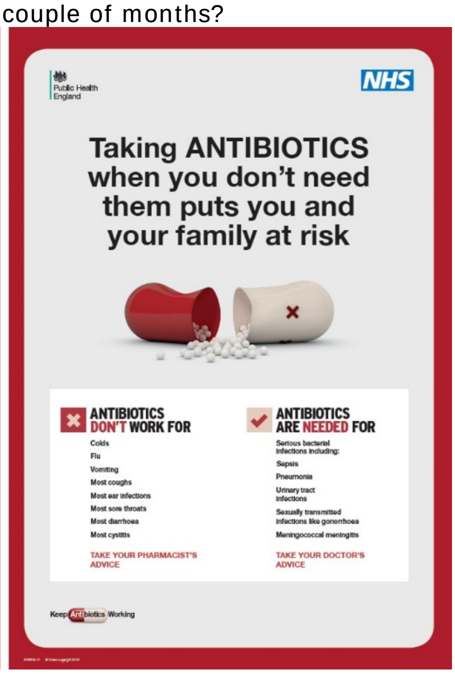

Show picture(s): Show poster image and digital display ad

Normal

1	Remember seeing these ads – in the last couple of months
5	Remember seeing these ads – longer ago
6	Not these ads, but something like them – in the last couple of months
2	Not these ads, but something like them – longer ago
3	Do not remember seeing these ads, or any like them **Exclusive*
4	Don't know **Exclusive*

Scripter notes: Do not allow 1 to be combined with 6 or 2
Do not allow 5 to be combined with 6 or 2

Ask only if **Q038 - NEW5**,1,5

Q039 - NEW6: **Multi coded**

Not back | Min = 1

And where did you see these ads?

Please select all that apply

Normal

1	In a newspaper or magazine
2	On an outdoor poster or billboard
3	In a pharmacy, GP surgery or other healthcare centre
4	In a school, nursery or children's centre
5	In a leaflet or advice sheet
6	On social media (e.g. Facebook)
7	On an NHS website (e.g. GP surgery website)
8	On a non-NHS website
9	Somewhere else (specify) **Open*
10	Don't know

**Q040 - NEW9:**	Matrix

Not back | Number of rows: 14 | Number of columns: 7

Here are some things people have said about the ads that you have just seen and heard. How much do you agree or disagree with each one?

Random

Rendered as Dynamic Grid

	Agree strongly	Tend to agree	Neither agree nor disagree	Tend to disagree	Strongly disagree	Not applicable	Don't know
These ads were aimed at people like me	❍	❍	❍	❍	❍	❍	❍
These ads really caught my attention	❍	❍	❍	❍	❍	❍	❍
These ads told me something new	❍	❍	❍	❍	❍	❍	❍
People are talking about these ads	❍	❍	❍	❍	❍	❍	❍
These ads are clear and easy to understand	❍	❍	❍	❍	❍	❍	❍
It is important that adverts like this are shown	❍	❍	❍	❍	❍	❍	❍
The advertising stands out from other advertising	❍	❍	❍	❍	❍	❍	❍
I am fed up of seeing this type of advertising	❍	❍	❍	❍	❍	❍	❍
These ads made me less likely to ask my GP for antibiotics	❍	❍	❍	❍	❍	❍	❍
These ads made me less likely to insist my GP prescribes antibiotics	❍	❍	❍	❍	❍	❍	❍
These ads made me think about the risks of taking antibiotics	❍	❍	❍	❍	❍	❍	❍
These ads made me consider searching online for more information on antibiotics	❍	❍	❍	❍	❍	❍	❍
These ads will make people I know less likely to insist their GP prescribe antibiotics	❍	❍	❍	❍	❍	❍	❍

**PRESOCIAL:**	**Text**

Not back

Next we will show you a short ad you may have seen online.

Q047 - NEWSOCIAL: | **Multi coded**

Not back | Min = 1

And have you seen this short video ad, or anything similar, online in the last couple of months?

Show picture(s):
Dr Ranj – Strong

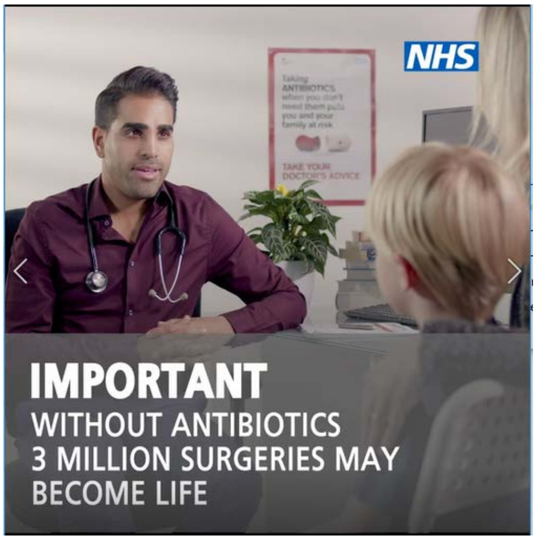

Normal

1 Yes, on Facebook
2 Yes, on Twitter
3 Yes, on Instagram
4 Yes, elsewhere **Open*
5 Yes, but not sure where **Exclusive*
6 Do not remember seeing this ad, or any like it **Exclusive*
7 Don’t know **Exclusive*

Q056 - SocialDiagnostics: Matrix

Not back | Number of rows: 14 | Number of columns: 7

Here are some things people have said about the video ads that you have just seen and heard. How much do you agree or disagree with each one?

Random

Rendered as Dynamic Grid

	Agree strongly	Tend to agree	Neither agree nor disagree	Tend to disagree	Strongly disagree	Not applicable	Don't know
These ads were aimed at people like me	❍	❍	❍	❍	❍	❍	❍
These ads really caught my attention	❍	❍	❍	❍	❍	❍	❍
These ads are clear and easy to understand	❍	❍	❍	❍	❍	❍	❍
It is important that adverts like this are shown	❍	❍	❍	❍	❍	❍	❍
The advertising stands out from other advertising	❍	❍	❍	❍	❍	❍	❍
I am fed up of seeing this type of advertising	❍	❍	❍	❍	❍	❍	❍
These ads made me less likely to ask my GP for antibiotics	❍	❍	❍	❍	❍	❍	❍
These ads made me less likely to insist my GP prescribes antibiotics	❍	❍	❍	❍	❍	❍	❍
These ads made me consider searching online for more information on antibiotics	❍	❍	❍	❍	❍	❍	❍

Q048 – Believability: Matrix

Not back | Number of rows: 5 | Number of columns: 6

How believable do you think each of the below statements is?

Rows: Random | Columns: Normal

Rendered as Dynamic Grid

	Very believable	Quite believable	Not very believable	Not believable at all	Don't know **Position fixed *Exclusive*
In 30 years, antibiotic resistance could kill more people than cancer and diabetes combined	❍	❍	❍	❍	❍
If antibiotics stop working, common surgeries such as caesareans may become life-threatening	❍	❍	❍	❍	❍
If you take antibiotics when you don’t need them, they may not work when you really do	❍	❍	❍	❍	❍
[IF PARENT] Antibiotic resistance is a threat to your child’s future	❍	❍	❍	❍	❍

Q041 - NEW7:	Single coded

Not back

There has also been media coverage about antibiotics, from Public Health England, in the last few months. This has been on TV programmes, newspapers, magazines and websites.

Here are some examples ... [INCLUDE JPG EXAMPLES OF PR COVERAGE]

Have you seen or heard any of this media coverage about Antibiotics in the last couple of months?

Please think of media coverage, not the advertising we showed you earlier.

Normal

1	Yes – in the last couple of months
4	Yes – longer ago
2	No
3	Don't know

Ask only if **Q035 - NEW2**,1,4 or **Q037 - NEW4**,1,4 or **Q038 - NEW5**,1,6,5,2 or **Q041 - NEW7**,1 or **Q047 – NEWSOCIAL**,1,2,3,4,5

Q049 - HotState	Multi coded

Not back | Min = 1

As a direct result of seeing these ads or media, did you do any of the following? Please choose all that apply.

Random

1	I didn’t ask my GP to prescribe antibiotics when I thought I needed them
2	[IF PARENT] I didn’t ask my GP to prescribe antibiotics for my child when I thought I needed them
3	I didn’t expect to be given antibiotics when I went to see my GP
4	[IF PARENT] I didn’t expect to be given antibiotics for my child when I went to see my GP
5	I didn’t insist my GP prescribe antibiotics when they said they weren’t needed
6	[IF PARENT] I didn’t insist my GP prescribe antibiotics for my child when they said they weren’t needed
7	I went to the pharmacist instead of going to my GP
8	I went to another non-urgent NHS service instead of going to my GP (such as a walk-in centre)
9	Called NHS 111 for further advice or information
10	None of the above **Position fixed *Exclusive*
11	Don't know **Position fixed *Exclusive*

Scripter notes:
Group and randomise within:
1,2
3,4
5,6

Ask only if **Q035 - NEW2**,1,4 or **Q037 - NEW4**,1,4 or **Q038 - NEW5**,1,6,5,2 or **Q041 - NEW7**,1 or **Q047 – NEWSOCIAL**,1,2,3,4,5

Q050 - Coldstate: | **Multi coded**

Not back | Min = 1

And again, as a direct result of seeing these ads or media, did you do any of these things? Please choose all that apply.

Random

1	I spoke to my GP about antibiotic resistance
2	I spoke to a friend or family member about antibiotic resistance
3	I spoke to my pharmacist about antibiotic resistance
4	I spoke to another healthcare professional about antibiotic resistance
5	Visited an NHS website for further advice or information
6	Visited a website other than the NHS for further advice or information
7	Looked for more information on Facebook, Twitter or other social media
8	Shared information or a comment on Facebook, Twitter or other social media
9	I thought differently about the use of antibiotics
10	Something else (specify) **Open *Position fixed*
11	None of the above **Position fixed *Exclusive*
12	Don't know **Position fixed *Exclusive*

Scripter notes:
Group 1,2,3,4, randomise within and keep 4 at the end
Group 5,6 always in this order
Group 7 and 8 and randomise within

Q043 - NEW10: | **Open**

Not back

Thinking about all the advertising you have just seen, what, if anything, **will** you do as a direct result of the campaign?

Random

1	I will always take my doctor's advice about antibiotics
2	I will take the advice given in the ads
3	I will talk to friends / family / others about antimicrobial resistance
4	I will only take antibiotics when I need them
5	I will not ask my doctor for antibiotics
6	I will trust my doctor to make the right decision
7	I will only take antibiotics that have been prescribed to me, not to someone else
8	I will look for information about antimicrobial resistance online
9	I will talk to my GP / other healthcare
13	I won't do anything **Position fixed *Exclusive*
14	Something else (specify) **Open *Position fixed*
15	Don't know**Open *Position fixed*

Scripter notes:

If Q043 – NEW10 = 13

**Q046 – NEW22:**	**Open**

Not back

You said you won't do anything as a result of seeing the ads. Why is that?

Random

1	I am already doing the right thing / I don't ask for antibiotics
2	I rarely go to the doctor's
3	I only see my doctor when I am very ill
4	I already knew about the dangers of taking unnecessary antibiotics / antimicrobial resistance
5	I haven't done anything / I won't do anything
8	It is my GP's responsibility to stop prescribing unnecessary antibiotics
6	Other reason (specify)
7	Don't know

Ask only if Q010 – Q039 statement 1 or 2 = 2-4 (if visited their GP in the last year / prescribed antibiotics)

**Q044 - NEW20:**	**Single coded**

Not back

You said that you recently spoke to your GP about antibiotics - during this conversation were you given this Treating Your Infection advice sheet, or one like it?

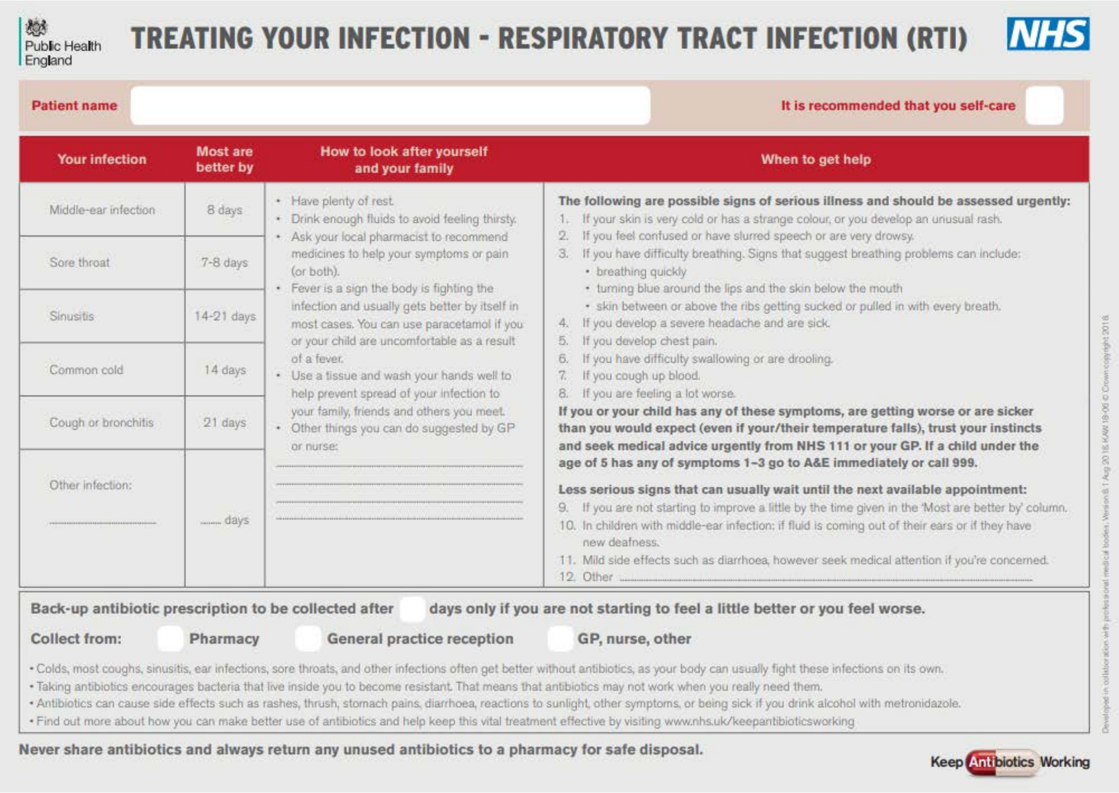
Public Health England

TREATING YOUR INFECTION - RESPIRATORY TRACT INFECTION (RTI)

NHS

Patient name

It is recommended that you self-care

Your infection	Most are better by	How to look after yourself and your family	When to get help
Middle-ear infection	8 days	• Have plenty of rest.<br>• Drink enough fluids to avoid feeling thirsty.<br>• Ask your local pharmacist to recommend medicines to help your symptoms or pain (or both).<br>• Fever is a sign the body is fighting the infection and usually gets better by itself in most cases. You can use paracetamol if you or your child are uncomfortable as a result of a fever.<br>• Use a tissue and wash your hands well to help prevent spread of your infection to your family, friends and others you meet.<br>• Other things you can do suggested by GP or nurse:	**The following are possible signs of serious illness and should be assessed urgently:**<br>1. If your skin is very cold or has a strange colour, or you develop an unusual rash.<br>2. If you feel confused or have slurred speech or are very drowsy.<br>3. If you have difficulty breathing. Signs that suggest breathing problems can include:<br>• breathing quickly<br>• turning blue around the lips and the skin below the mouth<br>• skin between or above the ribs getting sucked or pulled in with every breath.<br>4. If you develop a severe headache and are sick.<br>5. If you develop chest pain.<br>6. If you have difficulty swallowing or are drooling.<br>7. If you cough up blood.<br>8. If you are feeling a lot worse.<br>**If you or your child has any of these symptoms, are getting worse or are sicker than you would expect (even if your/their temperature falls), trust your instincts and seek medical advice urgently from NHS 111 or your GP. If a child under the age of 5 has any of symptoms 1–3 go to A&E immediately or call 999.**<br>**Less serious signs that can usually wait until the next available appointment:**<br>9. If you are not starting to improve a little by the time given in the 'Most are better by' column.<br>10. In children with middle-ear infection: if fluid is coming out of their ears or if they have new deafness.<br>11. Mild side effects such as diarrhoea, however seek medical attention if you're concerned.<br>12. Other ....
Sore throat	7-8 days		
Sinusitis	14-21 days		
Common cold	14 days		
Cough or bronchitis	21 days		
Other infection:	...... days		

Back-up antibiotic prescription to be collected after ☐ days only if you are not starting to feel a little better or you feel worse.

Collect from: ☐ **Pharmacy** ☐ **General practice reception** ☐ **GP, nurse, other**

• Colds, most coughs, sinusitis, ear infections, sore throats, and other infections often get better without antibiotics, as your body can usually fight these infections on its own.
• Taking antibiotics encourages bacteria that live inside you to become resistant. That means that antibiotics may not work when you really need them.
• Antibiotics can cause side effects such as rashes, thrush, stomach pains, diarrhoea, reactions to sunlight, other symptoms, or being sick if you drink alcohol with metronidazole.
• Find out more about how you can make better use of antibiotics and help keep this vital treatment effective by visiting www.nhs.uk/keepantibioticsworking

Never share antibiotics and always return any unused antibiotics to a pharmacy for safe disposal.

Keep Antibiotics Working

Developed in collaboration with professional medical bodies. Version 8.1 Aug 2018. KAW 18-06 © Crown copyright 2018

Normal

1	Yes
2	No
3	Don't know

SCRIPTER: Show image of TYI pad

Ask only if **Q044 - NEW20**,1

**Q045 - NEW21:**	Single coded

Not back

And how useful, if at all, did you find the Treating Your Infection advice sheet?

Normal

1	Very useful
2	Quite useful
3	Not very useful
4	Not at all useful
5	Don't know

**Q029 - Q049: Long term conditions**	Single coded

Not back

Are your day to day activities limited because of a health problem or disability which has lasted (or is expected to last) longer than 12 months?

Normal

1	Yes - limited a lot
2	Yes - limited a little
3	No
97	Prefer not to say **Position fixed *Exclusive*

**Q030 - Q030_Copy_1: Prescription payment**	Single coded

Not back

Do you pay for your prescription medicine?

Normal

1	Yes
2	No
99	Don't know **Position fixed *Exclusive*

Q031 - Q026: Ethnicity | Single coded

Not back

Please note that the following question is purely for market research purposes. The information you provide is anonymous and treated with utmost confidentiality. The data collected enables us to ensure we cover a fair representation of the population. Which of the following ethnic group or groups do you most identify with?

Normal

1	British
2	Irish
3	Any other White background
4	White and Black Caribbean
5	White and Black African
6	White and Asian
7	Any other Mixed background
8	Indian
9	Pakistani
10	Bangladeshi
11	Any other Asian background
12	Caribbean
13	African
14	Any other Black background
15	Chinese
16	Any other ethnic group
17	Prefer not to say

Q032 - Q032_Copy_1: | Single coded

Not back

Our client PHE would like to analyse the results of this survey using geographical areas. Would you agree to share your postcode with PHE for that purpose?

Normal

1	Yes, I agree to share my postcode with PHE for this purpose
2	No, I do not agree

Scripter notes: Scripter notes: Please script the two text boxes next to the 'Please enter your postcode' response

Q033 - Q033_Copy_2: | Single coded

Not back

Thank you very much for participating in this survey which is on behalf of Public Health England. Sometimes we (Kantar) want to re-contact respondents about a survey to ask additional questions on this topic, or to investigate your opinions on other future research for Public Health England. Would you agree for us to re-contact you in the next 12 months for this purpose?

Normal

1	Yes, I agree
2	No, I do not agree

Scripter notes: If code 1 = Yes --> script standard follow-up to capture contact details

Supplementary Material 3. CATI questionnaire

CATI Questionnaire

Name of survey
AMR 2019 post stage GP questionnaire

Client name
Public Health England

Author(s)
Rachel Feechan

This questionnaire was written according to Kantar quality procedures

checked by
Tori Harris

Repeating study (if this survey has been previously conducted)	
Name of survey	AMR 2019 GP questionnaire
Language	English (United Kingdom)
Survey length (minutes)	9
Version	2
Author(s)	Rachel Feechan<br>Tori Harris
Contact	
Panel	
Sample size	Gross: 300<br>Net:
Sample description	GPs (100 in Granada, 200 in rest of England)
Quota	English region (North, Mids, South)<br>Size of surgery (1-5 and 6+ GPs)
If several countries: indicate the countries	
If several targets	
Check-in site	http://www.kantar.com
Comments	

Index

Q001 - Q001: Intro
Q002 - Q002_1: Frequency of being inappropriately asked for ABs
Q003 - Q003_1: Inappropriate prescription demands compared with last year
Q004 - Q004_1: Actions taken when inappropriately asked for antibiotics
Q005 - Q005_NEW: Q5NEW: Last actions taken when inappropriately asked for antibiotics
Q006 - Q006_1: Perceptions of pressure to prescribe among GPs
Q007 - Q7New: Attitudes re antibiotic prescription
Q008 - Q008_1: Levels of patient awareness
Q009 - Q009_1:
Q010 - Q010_1: Media awareness
Q014 - NEW1:
Q015 - NEW2:
Q016 - NEW3:
Q017 - NEW4:
Q011 - Q011_1: Years been a GP
Q012 - Q012: Close
Q013 - Q013_1: Gender

Q001 - Q001: Intro **Text**

Not back

Good morning/afternoon/evening. My name is..........and I am calling on behalf of Kantar ~~Public~~, an independent research company. We have been commissioned by Public Health England to conduct a survey amongst GPs about prescription of antibiotics. Please can I speak to [NAMED RESPONDENT]?

IF YES - GO TO 'WHEN THROUGH TO GP' ON THE NEXT SCREEN

IF NO, SAY:

Are there any other GPs available to speak to?

IF YES - GO TO 'WHEN THROUGH TO GP' ON THE NEXT SCREEN

IF NO, OR IF ASKED FOR REASSURANCE OR IF TOLD 'DON'T DO RESEARCH' THEN SAY:

Would you like me to send you through an email from Public Health England to reassure you of the nature of this research? We would very much appreciate it if you are able to take part.

IF NO – END CALL

IF YES – ARRANGE TO SEND RE-ASSURANCE EMAIL AFTER COLLECTING EMAIL ADDRESS

IF ASKED:

The survey will take around 9 minutes.

IF NAMED GP IS UNAVAILABLE AT THAT POINT BUT A LATER TIME SLOT IS OFFERED COLLECT DETAILS FOR A LATER CALL

WHEN THROUGH TO GP:

Good morning/afternoon. My name is..........and I am calling on behalf of Kantar ~~Public~~, an independent research company.

We have been commissioned by Public Health England to conduct a survey amongst GPs about their thoughts about prescribing antibiotics.

The survey will take around 9 minutes. Is now a convenient time?

WHEN THEY HAVE AGREED TO TAKE PART:

I just want to reassure you that this is confidential, voluntary social research. I would like to inform you that for quality control and training purposes, the interview might be monitored or recorded.
Thank you for agreeing to participate.

Q002 - Q002_1: Frequency of being inappropriately asked for ABs | Single coded

Not back

How often, if at all, do you get asked by patients to prescribe antibiotics when you have said they aren't needed?

READ OUT EACH STATEMENT AND SELECT AS APPROPRIATE

Normal

1	Very frequently
2	Fairly frequently
3	Occasionally
4	Rarely
5	Never
6	Don't know (DO NOT READ OUT)

Scripter notes: Codes 1-5 should be flipped for 50% of respondents.

Q018 - Q018_1: Frequency of being inappropriately asked for ABs for child | Single coded

Not back

How often, if at all, do you get asked by patients to prescribe antibiotics **for their child** when you have said they aren't needed?

READ OUT EACH STATEMENT AND SELECT AS APPROPRIATE

Normal

1	Very frequently
2	Fairly frequently
3	Occasionally
4	Rarely
5	Never
6	Don't know (DO NOT READ OUT)

Scripter notes: Codes 1-5 should be flipped for 50% of respondents.

Ask only if **Q002 - Q002_1**,1,2,3,4 or **Q018 - Q018_1**,1,2,3,4

**Q004 - Q004_1: Actions taken when inappropriately asked for antibiotics**	**Multi coded**

Not back

Thinking about those times in the last couple of months when you have been asked by patients for antibiotics when you don't think they are needed, have you ever done any of the following?

READ OUT EACH STATEMENT AND SELECT AS APPROPRIATE

Random

1	Given the patient a letter or leaflet explaining why they don't need antibiotics on this occasion
2	Given the patient a delayed prescription for antibiotics
3	Explained the issue of antimicrobial resistance to the patient
4	Explained the medical reasons why antibiotics are not appropriate e.g. they are for bacterial infections rather than viral
5	Re-assured them that their symptoms will clear soon without antibiotics
6	Prescribed antibiotics
7	Given self-care advice
9	Offered a follow-up appointment if the patient no better/still concerned
10	Used the “Treat your infection” leaflet to explain self-care and the role of antibiotics ~~Given the patient a ‘Treat your infection’ pad/leaflet~~
8	Something else? **Open *Position fixed*
98	None of the above **Position fixed *Exclusive*

Scripter notes: Randomise codes 1-10 (excluding code 8)

Ask only if **Q002 - Q002_1**,1,2,3,4 or **Q018 - Q018_1**,1,2,3,4

**Q005 - Q005_NEW: Q5NEW: Last actions taken when inappropriately asked for antibiotics**	**Multi coded**

Not back

And thinking about the **last time** that you were asked by a patient for antibiotics when you didn't think they were needed, which if any of the following did you do?

READ OUT EACH STATEMENT AND SELECT AS APPROPRIATE

Random

1	Gave the patient a letter or leaflet explaining why they don't need antibiotics on this occasion
2	Gave the patient a delayed prescription for antibiotics
3	Explained the issue of antimicrobial resistance to the patient
4	Explained the medical reasons why antibiotics are not appropriate e.g. they are for bacterial infections rather than viral
5	Re-assured them that their symptoms will clear soon without antibiotics
6	Prescribed antibiotics
7	Gave self-care advice
9	Offered a follow-up appointment if the patient no better/still concerned
10	Used the “Treat your infection” leaflet to explain self-care and the role of antibiotics ~~Given the patient a ‘Treat your infection’ pad/leaflet~~
8	Something else? **Open *Position fixed*
98	None of the above **Position fixed *Exclusive*

Scripter notes: Randomise codes 1-10 (excluding code 8)
Please randomize in same order as Q004
Bold “Last time” in question text

Q006 - Q006_1: Perceptions of pressure to prescribe among GPs | Single coded

Not back

In general, how much pressure, if any, do you feel from your patients to prescribe antibiotics?

READ OUT EACH STATEMENT AND SELECT AS APPROPRIATE

Normal

1	A great deal of pressure
2	A fair amount of pressure
3	A little pressure
4	No pressure
5	Don't know (DO NOT READ OUT) **Position fixed*

Scripter notes: Codes 1-4 to be flipped for 50% respondents

Ask only if **Q004 - Q004_1**,6 or **Q005 - Q005_NEW**,6

Q023 - WhyPrescribed | Open

Not back

You said that you have prescribed antibiotics to a patient when you didn't think they were needed. What do you think are some reasons for this?

IF NECESSARY: Anything else?

Interviewer notes: Could include things such as:

- Did not have enough time within appointment to explain to the patient
- I knew the patient(s) wouldn't understand/accept it even if I explained
- I knew they would come back and ask again
- Felt pressured by the patient
- Felt pressured because the patient was a child
- I didn't want to risk it just in case it was a bacterial infection
- Prescribing antibiotics is the safer option
- They were probably going to go find antibiotics elsewhere if I didn't prescribe it

Q007 - Q7New: Attitudes re antibiotic prescription | **Matrix**

Not back | Number of rows: 8 | Number of columns: 5

For each of the following statements, please tell me whether you strongly disagree, disagree, agree, or strongly agree...

READ OUT EACH STATEMENT AND SELECT AS APPROPRIATE

Random

Rendered as Dynamic Grid

	Strongly disagree	Disagree	Agree	Strongly agree	Don't know (DO NOT READ OUT)
Patients generally accept my decision if I decline to prescribe antibiotics	❍	❍	❍	❍	❍
If I decline to prescribe antibiotics when a patient asks for them I worry that the patient’s clinical condition might worsen	❍	❍	❍	❍	❍
I am confident I can say no to most patients asking for antibiotics when I don’t think they are needed	❍	❍	❍	❍	❍
I am confident I can say no to all patients asking for antibiotics when I don’t think they are needed	❍	❍	❍	❍	❍
I am confident my GP colleagues say no to all patients asking for antibiotics when they don’t think they are needed	❍	❍	❍	❍	❍
All GPs regularly prescribe antibiotics when they think it will reassure a worried patient	❍	❍	❍	❍	❍
There is good support available for GPs who want to reduce the amount of antibiotics they prescribe	❍	❍	❍	❍	❍
Antimicrobial Resistance is a serious issue facing the NHS today	❍	❍	❍	❍	❍
Patients are less likely to expect me to prescribe them antibiotics than this time last year	❍	❍	❍	❍	❍

Scripter notes: STATEMENT 3: PLEASE EMBOLDEN "MOST"
STATEMENT 4 : PLEASE EMBOLDEN "ALL"
STATEMENT 5: PLEASE EMBOLDEN "ALL"
Please flip codes 1-4 for 50% of respondents
Group statements 3,4,5 and keep in this order

Q008 - Q008_1: Levels of patient awareness | Single coded

Not back

And if you had to guess, how many of your patients would you say fully understand the risk of antimicrobial resistance as a result of taking antibiotics when they are not needed? Please use a scale of 0 to 10 where 0 is none of them and 10 is all of them.

DO NOT READ OUT ANSWER CODES

Normal

1	0
2	1
3	2
4	3
5	4
6	5
7	6
8	7
9	8
10	9
11	10
12	Don't know (DO NOT READ OUT)

Q009 - Q009_1: | Single coded

Not back

Have you seen or heard any media or publicity about anti-microbial resistance (often called AMR) or levels of inappropriate prescription in the last six weeks or so?

DO NOT READ OUT

Normal

1	Yes
2	No
99	Don't know **Position fixed *Exclusive*

**Q014 - NEW1:**	**Single coded**

Not back

There has recently been an advertising campaign featuring radio and TV adverts, press and posters about antibiotics. These ads include images of red and white pill capsules, with messages including:
‘taking antibiotics for the wrong things is dangerous to do’;
‘‘taking antibiotics when you don’t need them puts you and your family at risk’ and
‘always take your doctor’s advice’.

Some ads also include messages around antibiotic resistance making common operations life threatening in future and that it could kill more people than cancer and diabetes.

Have you seen or heard this campaign?

If Yes, probe for when seen

Normal

1	Yes – in the last couple of months
4	Yes – seen before that
2	No
3	Not sure/Don't know

SCRIPTER NOTES: Allow multicode of codes 1 and 4.

Ask only if **Q014 - NEW1**,1,4

Q015 - NEW2: **Multi coded**

Not back | Min = 1

And where have you seen or heard this campaign?

DO NOT READ OUT, CODE ALL THAT APPLY

Normal

1 Something in a specialist GP magazine
2 TV advertising
3 Radio advertising
4 Local newspaper advertising
5 Internet advertising
6 Leaflet
7 Posters
8 Something in your GP surgery
9 National press advertising
10 Pharmacy bags advertising
26 Something in your local community (e.g. in a pharmacy or dentist surgery)
11 Media coverage (e.g. TV programme/radio programme/press article etc.)
12 Heard about it from friends/family
13 Heard about it from a colleague
14 Events in public places
15 Something a patient showed me
16 Something on a website
17 Something on social media
18 Something from the local CCG/SCN (Clinical Commissioning Group/ Strategic Clinical Network)
19 Something from Public Health England (PHE)/ Department of Health (DH)/ NHS England/ Chief Medical Officer
20 Something from a professional group (e.g. Royal College of GPs/ British Medical Association)
25 Via the Antibiotic Guardian campaign (website, email, social media)
21 A toolkit/letter/posters/pad from Public Health England / ~~Paul Cosford~~
22 Anything else (other specify) *Open
23 Don’t know
24 Refused

Ask only if **Q014 - NEW1**,1,4

Q016 - NEW3: **Matrix**

Not back | Number of rows: 6 | Number of columns: 6

Now, still thinking about this advertising, please tell me whether you strongly disagree, disagree, neither agree nor disagree, agree, or strongly agree with the following statements...

READ OUT EACH STATEMENT AND SELECT AS APPROPRIATE

Normal

	Strongly disagree	Disagree	Neither agree nor disagree	Agree	Strongly agree	Don't know (DO NOT READ OUT)
It is important that advertising like this is shown	❍	❍	❍	❍	❍	❍
The advertising will make patients less likely to ask for antibiotics when you say they aren't needed	❍	❍	❍	❍	❍	❍
The advertising will make me more confident to say no to patients asking for antibiotics when I think they aren't needed	❍	❍	❍	❍	❍	❍
The advertising will help to raise awareness of the issue of antimicrobial resistance	❍	❍	❍	❍	❍	❍
The advertising supports GPs to say no to patients asking for antibiotics when they think they are not needed	❍	❍	❍	❍	❍	❍
The advertising supports GPs to discuss with their patients reasons why they may not prescribe antibiotics	❍	❍	❍	❍	❍	❍
The advertising supports me to achieve targets around reducing inappropriate prescription of antibiotics	❍	❍	❍	❍	❍	❍

Scripter notes: Randomise statements, but always show 1-4 before 5 and 6.

Q017 - NEW4: **Single coded**

Not back

Public Health England recently sent all GP surgeries a GP AMR toolkit pack, including three pads of 'Treating your infection' respiratory tract infection (RTI) advice sheets for use in consultations. The pack also included 'Keep Antibiotics Working' posters, advice sheets and target leaflets, and a leaflet dispenser to help advise patients about how best to look after themselves and their family if antibiotics aren't needed and how to recognise if they are.

~~Public Health England recently sent all GP surgeries a GP AMR toolkit pack, including a 'treating your infection' respiratory tract infection RTI advice sheet for use in consultations. The pack also included posters, advice sheets and target leaflets informing patients about how best to look after themselves and their family if antibiotics aren't needed and how to recognise if they are.~~

How useful, if at all, did you find the pack in consultations with your patients?

READ OUT, CODE ONE.

Normal

1 Very useful
2 Quite useful
3 Not very useful
4 Not useful at all
5 I did not use this pad (do not read out) **Position fixed*
6 Don't know (DO NOT READ OUT) **Position fixed*

Q020 - UTIPadDownloads: **Multi-coded**

Not back

PHE also have another 'Treating Your Infection' information pad for use with patients in consultations – the 'Urinary Tract Infection UTI' pad.

Have you received this, or downloaded this, from the PHE Campaign Resource Centre?

Normal

1 Yes
2 No
3 Not sure/Don't know

ASK ALL WHO DOWNLOADED THE UTI PAD (UTIPadDownloads,1)

Q021 - UTIPadUseful: **Single coded**

Not back

Thinking specifically about the Urinary Tract Infection pad, how useful, if at all, did you find it in consultations with your patients?

READ OUT, CODE ONE.

Normal

1 Very useful
2 Quite useful
3 Not very useful
4 Not useful at all
5 I did not use this pad (do not read out)

ASK ONLY IF **Q021-UTIPadUseful**,1,2,3,4 OR **Q017-NEW4**,1,2,3,4

Q019- NEW6: Single coded

Not back

[V1]: And still thinking about the Urinary Tract Infection pad specifically, to what extent would you agree or disagree with these statements?

[V2]: Now thinking about the Respiratory Tract Infection RTI pad specifically, to what extent would you agree or disagree with these statements?

READ OUT AND READ OUT SCALE, REPEAT AS NEEDED

Random
SCRIPTER: If respondent says code 6 (I have not used the pad) at the first statement, go to next question.

	Strongly disagree	Disagree	Neither agree nor disagree	Agree	Strongly agree	I have not used the pad	Don't know (DO NOT READ OUT)
Patients find the information in the pad easy to understand	❍	❍	❍	❍	❍	❍	❍
It helps to reassure patients	❍	❍	❍	❍	❍	❍	❍
It helps patients understand how to look after themselves if they feel unwell	❍	❍	❍	❍	❍	❍	❍
It makes patients less likely to insist for antibiotics when you say they aren't needed	❍	❍	❍	❍	❍	❍	❍
It makes you more confident to say no to patients asking for antibiotics when you think they aren't needed	❍	❍	❍	❍	❍	❍	❍

SCRIPTER NOTES: Each respondent sees only one version of Q019.
If **Q021-UTIPadUseful**,1,2,3,4 show V1
If **Q017-NEW4,**1,2,3,4 AND (**Q020 – UTIPadDownloads**,2,3 OR **Q021-UTIPadUseful**,5) show V2
If **Q017-NEW4,**1,2,3,4 AND **Q021-UTIPadUseful**,1,2,3,4 show V1

Q022 - HelpfulforGPs Open

Not back

Besides the 'Treating Your Infection' RTI and UTI pads, what else do you think would be helpful for you to reduce inappropriate prescribing?

IF NECESSARY: Anything else?

Q011 - Q011_1: Years been a GP | **Numeric**

Not back | Max = 99

And finally, to help us classify your experience, please could you let me know how many years have you been a GP?
ENTER NUMBER (0-99)
INTERVIEWER NOTE: IF LESS THAN A YEAR CODE ZERO

IF RESPONDENT UNSURE: Please just estimate to the best of your recollection.

Q012 - Q012: Close | **Single coded**

Not back

Being part of the Kantar group, from time to time we need to interview people who meet certain criteria for specific research surveys. As you have been so helpful today, would you be willing for the Kantar Group to keep a record of your details for the purpose of re-contacting you on behalf of PHE to take part in future research within the next 24 months?

Normal

1	Yes
2	No

Scripter notes: PLEASE PROVIDE A BOX FOR THE INTERVIEWER TO ENTER THE NAME OF THE DOCTOR IF YES IS SELECTED

Q013 - Q013_1: Gender | **Single coded**

Not back

DO NOT READ OUT - INTERVIEWER PLEASE CODE SEX OF RESPONDENT

Normal

1	Male
2	Female
3	Don't know

Supplementary Material 4a. Table showing unprompted campaign recognition among the public and campaign target groups and mode of campaign recognition, England 2017 to 2019

			Pre 2017		Post 2017		Pre 2018		Post 2018		Pre 2019		Post 2019			
			n	%	*n*	%	*n*	%	*n*	%	*n*	%	*n*	%	X^2	P-value
Un-prompted awareness of any AMR publicity	All (*n* = 1000)	Yes	126	12.6	335	33.5	148	14.8	243	24.3	171	17.1	252	25.2	52.263	<0.001
		No	807	80.7	592	59.2	773	77.3	682	68.2	755	75.5	678	67.8		
		Don't Know	67	6.7	74	7.4	79	7.9	75	7.5	75	7.5	70	7.0		
	Mothers of children aged 0-16	Yes	21	16.9	51	38.9	20	15.3	37	28.2	28	21.4	41	31.3	8.919	0.003
		No	90	72.6	80	61.1	100	76.3	83	63.4	91	69.5	81	61.8		
		Don't Know	13	10.5	0	0.0	11	8.4	10	7.6	12	9.2	9	6.9		
		Total	124	100	131	100	131	100	131	100	131	100	131	100		
	Adults aged over 50-yrs	Yes	37	8.8	148	34.7	53	12.9	77	19.0	52	12.5	80	19.3	19.632	<0.001
		No	357	84.8	245	57.2	323	78.0	329	81.0	364	87.5	335	80.7		
		Don't Know	27	6.4	35	8.1	38	9.1	0	0.0	0	0.0	0	0.0		
		Total	421	100	428	100	414	100	406	100	416	100	415	100		

AMR, antimicrobial resistance; GP, general practitioner; n = sample size; Unprompted recognition, participant recall of a campaign without the help of suggestions from interviewers; X^2, pearson's cumulative test statistic and p-value calculated using pre-2017 and post-2019 data

Supplementary Material 4b. Table showing prompted campaign recognition among the public, campaign target groups and GPs, England 2017 to 2019

			Post 2017		Post 2018		Post 2019			
			n	%	*n*	%	n	%	X^2	P-value
Promoted campaign recognition	All (*n*=1000)	Yes	NC	NA	680	68.0	740	74.0	8.742	0.003
		No	NC	NA	320	32.0	260	26.0		
	Mother of children aged 0-16-years (*n*=131)	Yes	NC	NA	91	69.5	109	83.2	9.283	0.002
		No	NC	NA	40	30.5	22	16.8		
	Adults aged over 50-years	Yes	NC	NA	283	69.7	298	71.8	0.469	0.493
		No	NC	NA	123	30.3	117	28.2		
		Total	NC	NA	406	100	415	100		
	GPs	Yes	184	63.7	178	62.0	205	60.1	0.835	0.361
		No	105	36.3	109	38.0	136	39.9		
		Total	289	100	287	100	341	100		

GP, general practitioner; *n*, sample size; NA, not applicable; NC, data not collected at this timepoint; prompted recognition, participant recall of a campaign with help of suggestions from interviewers, i.e the participant is shown campaign material and asked if they recognise it. X^2, Pearson's cumulative test statistic and p-value calculated using post-2018 and post 2019 data for all participants, mothers of children aged 0-16-yrs and adults aged over 50-yrs. Data from post-2017 to post-2019 used for GPs.

Supplementary Material 5. Table showing mode of campaign recognition, England 2017 to 2019

		Pre 2017		Post 2017		Pre 2018		Post 2018		Pre 2019		Post 2019	
		n	%	*n*	%	*n*	%	*n*	%	*n*	%	*n*	%
How the participant became aware of the campaign	TV programme or advert	42	33.4	265	79.1	61	41.0	170	70.0	75	44.2	153	60.7
	Newspaper article or advert	25	19.9	72	21.5	40	27.1	25	10.4	35	20.5	42	16.7
	Magazine article or advert	16	12.7	32	9.5	19	12.7	9	3.7	16	9.5	20	7.9
	Radio	16	12.7	59	17.5	19	13.1	30	12.1	21	12.4	29	11.5
	Poster or leaflet	25	8.8	49	14.7	17	11.8	20	8.3	18	10.7	34	13.5
	Internet	24	19.0	46	13.8	40	26.0	34	14.1	41	23 .7	60	23.8
	Friend or relative	11	8.7	15	4.5	10	6.8	5	2.2	14	8.2	18	7.1
	Social media	27	21.5	33	9.9	25	17.3	39	16.0	28	17.0	53	21.0
	GP or GP surgery	45	35.7	55	37.2	53	35.9	54	22.2	45	26.3	14	5.6
	Pharmacist or at a pharmacy	25	8.8	42	12.7	30	20.1	23	9.6	35	20.8	65	25.8
	Other	8	6.4	2	0.6	5	3.1	2	0.8	7	3.7	27	10.7
	Don't Know	2	1.6	7	2.1	3	2.3	3	1.3	6	3.4	3	1.2
	Total	126	100	335	100	148	100	243	100	171	100	252	100

GP, general practitioner; TV, television; *n* = number of respondents

Supplementary Material 6. Descriptive statistics of perceived knowledge of antimicrobial and antibiotic resistance pre- and post-campaign by gender, socioeconomic status, and ethnicity, England 2017-2019

			Pre 2017								Post 2019									
			Know a lot		Know something		Heard of but know nothing about		Never heard of		Know a lot		Know something		Heard of but know nothing about		Never heard of		X^2	P-value
			n	%	*n*	%	*n*	%	*n*	%	*n*	%	*n*	%	*n*	%	*n*	%		
Antibiotic Resistance	All		84	8.7	477	49.3	320	33.1	86	8.9	101	10.4	540	55.7	254	26.2	74	7.6	13.952	0.003
	Sex	Male	42	8.7	228	47.1	166	34.3	48	9.9	43	9.1	274	58.3	117	24.9	36	7.7	14.223	0.003
		Female	42	8.7	249	51.4	154	31.8	39	8.1	58	11.6	266	53.3	137	27.5	38	7.6	3.899	0.273
	SES	ABC1	60	11.2	271	50.4	164	30.5	43	8.0	65	12.1	306	57.0	124	23.1	42	7.8	7.889	0.048
		C2DE	23	5.4	207	48.3	156	36.4	43	10.0	36	8.3	234	54.2	130	30.1	32	7.4	8.484	0.037
	Ethnicity	White	74	8.2	445	49.6	299	33.3	79	8.8	90	10.2	493	55.9	235	26.6	64	7.3	13.136	0.004
		Non-white	10	14.5	32	46.4	20	29.0	7	10.1	10	12.5	43	53.8	16	20.0	11	13.8	2.146	0.543
Anti-microbial Resistance	All		43	4.5	179	18.9	273	28.9	451	47.7	48	5.0	264	27.6	257	26.9	387	40.5	20.219	<0.001
	Sex	Male	29	6.1	90	19.0	153	32.3	202	42.6	25	5.4	151	32.5	123	35.6	165	35.6	22.623	<0.001
		Female	14	3.0	89	18.9	120	25.4	249	52.8	23	4.7	113	23.0	134	27.2	222	45.1	6.948	0.074
	SES	ABC1	27	5.1	107	20.4	156	29.7	235	44.8	35	6.6	150	28.1	145	27.2	203	38.1	10.907	0.012
		C2DE	16	3.8	72	17.1	118	28.0	216	51.2	14	3.3	114	27.0	112	26.5	183	43.3	12.502	0.006
	Ethnicity	White	39	4.4	161	18.3	252	28.7	426	48.5	40	4.6	244	28.0	231	26.5	356	40.9	24.174	<0.001
		Non-white	4	5.8	18	26.1	22	31.9	25	36.2	9	11.4	19	24.1	24	30.4	27	34.2	1.445	0.695

ABC1, Higher & intermediate managerial, administrative, supervisory, clerical & junior managerial and professional occupations; AMR, antimicrobial resistance; C2DE, Skilled manual occupations, semi-skilled & unskilled manual occupations, unemployed and lowest grade occupations; n = sample size; SES, socioeconomic status; X^2, Pearson's cumulative test statistic and p-value calculated using pre-2017 and post-2019 data

Supplementary Material 7. Descriptive statistics for the general public's knowledge, attitude and behaviour towards antimicrobial resistance and appropriate antibiotic usage across the three years of the campaign, England 2017-2019, (*n* = 1000)

		Pre 2017 (%)	Post 2017 (%)	Pre 2018 (%)	Post 2018 (%)	Pre 2019 (%)	Post 2019 (%)	X^2	P-value
People who thought colds are not treated with antibiotics		67.6	67.8	70.2	66.0	65.2	60.9	9.772	0.020
People who thought flu is not treated with antibiotics		55.6	55.5	56.0	52.7	55.2	48.3	10.096	0.010
Antibiotics don't work for everything	Strongly agree	57.3	63.3	59.8	62.9	64.5	60.1	6.171	0.013
	Agree	34.9	26.6	31.4	26.3	25.9	29.5		
	Disagree	3.1	4.2	3.6	5.0	4.4	5.3		
	Strongly disagree	1.4	1.6	1.6	1.7	1.8	1.9		
	Don't know	3.3	4.3	3.6	4.1	3.4	3.2		
Antibiotics will stop working for you if taken for the wrong things	Definitely true	22.5	28.9	30.2	32.7	33.6	35.0	5.753	0.016
	Probably true	46.6	47.2	47.1	44.7	45.5	42.6		
	Probably false	14.4	9.2	9.9	7.9	10.1	11.6		
	Definitely false	1.7	1.9	1.5	2.1	1.6	1.8		
	Don't know	14.8	12.7	11.2	12.5	9.3	9.1		
Taking antibiotics when you don't need them puts you and your family at risk of antibiotic resistant infections	Definitely true	NC	36.3	40.7	45.5	46.8	48.2	20.345	<0.001
	Probably true	NC	44.2	42.8	37.1	37.4	37.9		
	Probably false	NC	6.9	6.6	7.2	6.8	6.8		
	Definitely false	NC	1.5	1.7	1.1	1.7	1.4		
	Don't know	NC	11.0	8.2	9.1	7.3	5.8		
Antibiotics always speed up my recovery no matter what the illness	Strongly agree	5.9	5.6	6.5	4.9	7.8	8.0	0.553	0.457
	Agree	19.9	16.2	17.2	17.7	19.3	20.4		
	Disagree	32.0	33.1	31.0	30.1	29.4	28.9		
	Strongly disagree	30.3	31.1	31.9	35.7	33.6	33.7		
	Don't know	11.9	14.1	13.3	11.7	10.0	9.0		
Likelihood to ask your GP for antibiotics	Very likely	7.9	5.8	6.0	7.0	9.0	9.1	0.575	0.448
	Quite likely	12.4	10.6	13.4	11.7	11.8	12.9		
	Quite unlikely	27.8	24.6	24.6	21.9	23.3	23.5		
	Very unlikely	44.8	53.1	50.8	52.8	48.5	48.5		
	Don't know	7.0	5.9	5.2	6.7	7.3	6.0		
Hot state action: I did not expect to receive antibiotics	No	NC	NC	NC	87.3	NC	83.2	4.672	0.031
	Yes	NC	NC	NC	12.7	NC	16.8		

	Missing (*n*)	NC	NC	NC	345	NC	287		
Hot state action: Used another non-urgent NHS service	No	NC	NC	NC	97.0	NC	94.7	5.019	0.025
	Yes	NC	NC	NC	3.0	NC	5.3		
	Missing (*n*)	NC	NC	NC	345	NC	287		

GP, general practitioner; NC, data not collected at this timepoint; *n* = sample size; X^2, Pearson's cumulative test statistic and p-value calculated using pre-2017 and post-2019 data where available. For the variable "Taking antibiotics when you don't need them puts you and your family at risk of antibiotic resistant infections" post 2017 and post 2019 data was used, for hot state action variables post 2018 and post 2019 data was used.

Supplementary Material 8. Descriptive statistics for the parent's knowledge, attitude and behaviour towards antimicrobial resistance and their child's appropriate antibiotic usage across the three years of the campaign, England 2017-2019

		Pre 2017		Post 2017		Pre 2018		Post 2018		Pre 2019		Post 2019		X^2	P
		n	%	*n*	%	*n*	%	*n*	%	*n*	%	*n*	%		
I always take my GP's advice about whether my child needs antibiotics	Strongly agree	85	34.3	NC	NA	117	45.0	113	44.1	114	42.2	120	45.3	0.012	0.912
	Agree	128	51.6	NC	NA	116	44.6	111	43.4	122	45.2	112	42.3		
	Disagree	21	8.5	NC	NA	19	7.3	19	7.4	23	8.5	21	7.9		
	Strongly disagree	4	1.6	NC	NA	2	0.8	2	0.8	4	1.5	5	1.9		
	Don't know	10	4.0	NC	NA	6	2.3	11	4.3	7	2.6	7	2.6		
	Total	248	100	NC	NA	260	100	256	100	270	100	265	100		
I expect the GP to give me antibiotics for my child if I ask for them	Strongly agree	25	10.0	34	13.8	26	10.1	30	11.7	27	10.0	34	12.9	2.609	0.106
	Agree	71	28.4	62	25.1	63	24.4	57	22.3	81	29.9	88	33.3		
	Disagree	95	38.0	72	29.1	92	35.7	88	34.4	89	32.8	74	28.0		
	Strongly disagree	41	16.4	56	22.7	67	26.0	62	24.2	60	22.1	58	22.0		
	Don't know	18	7.2	23	9.3	10	3.9	19	7.4	14	5.2	10	3.8		
	Total	250	100	247	100	258	100	256	100	271	100	264	100		
Likelihood to ask your GP for antibiotics for others/your child	Very likely	56	15.0	51	13.4	13	9.9	11	8.4	14	10.7	21	16.0	0.480	0.488
	Quite likely	89	23.9	63	16.5	27	20.6	25	19.1	31	23.7	26	19.7		
	Quite unlikely	96	25.7	102	26.8	41	31.3	39	29.8	38	29.0	33	25.0		
	Very unlikely	107	28.7	142	37.3	45	34.4	45	34.4	37	28.2	43	32.6		
	Don't know	25	6.7	23	6.0	5	3.8	11	8.4	11	8.4	9	6.8		
	Total	373	100	381	100	131	100	131	100	131	100	132	100		

GP, general practitioner; IQR, interquartile range; NA, not applicable; NC, data not collected at this timepoint; *n* = sample size; X^2, Pearson's cumulative test statistic and p-value calculated using pre-2017 and post-2019 data

Supplementary Material 9. Descriptive statistics showing level of concern about antimicrobial resistance assessed using a 7-point Likert scale, England 2017-2019

		Pre 2017		Post 2017		Pre 2018		Post 2018		Pre 2019		Post 2019		Z-score	P-value
Concern about AMR (median (IQR)	For you	4	3-5	4	3-5	4	3-5	4	2-5	4	3-5	5	3-6	-5.091	<0.001
	For children	5	4-6	4	3-5	5	4-6	4	3-6	5	4-6	5	4-6	-3.616	<0.001

AMR, antimicrobial resistance; IQR, interquartile range; z-score and p-value calculated using pre-2017 and post-2019 data

Supplementary Material 10. GPs' attitudes towards the KAW campaign, GP prescribing attitudes and actions taken when inappropriately asked for antibiotics in GPs who have and have not seen the campaign, England 2017-2019

		2017						2018						2019					
		Aware of campaign (*n* = 184)		Unaware of campaign (*n* = 105)		X^2	P-value	Aware of campaign (*n* = 178)		Unaware of campaign (*n* = 109)		X^2	P-value	Aware of campaign (*n* = 206)		Unaware of campaign (*n* = 137)		X^2	P-value
		n	%	*n*	%			*n*	%	*n*	%			*n*	%	*n*	%		
I am confident I can say no to most patients asking for antibiotics when I don't think they are needed	Strongly agree	77	41.8	37	35.8	4.000	0.045	61	34.1	42	38.5	2.416	0.120	77	37.3	57	41.6	0.211	0.646
	Agree	105	57.0	60	57.1			113	63.9	64	58.7			115	56.0	73	53.3		
	Disagree	2	1.2	2	1.7			3	1.9	0	0.0			7	3.2	4	2.9		
	Strongly disagree	0	0	3	2.6			1	0.6	0	0.0			6	3.0	3	2.2		
	Don't know	0	0	3	2.8			0	0.0	3	2.8			1	0.6	0	0.0		
Explained the reasons why antibiotics are inappropriate	Yes	175	95.1	91	86.7	2.630	0.105	161	90.6	82	75.2	9.217	0.002	194	94.4	123	89.8	1.079	0.299
	No	9	4.9	10	9.5			17	9.4	24	22.0			11	5.6	11	8.0		
	Missing	0	0.0	4	3.8			0	0.0	3	2.8			0	0.0	3	2.2		
Explained the issue of AMR to the patient	Yes	145	78.6	72	71.5	2.028	0.154	127	71.7	66	62.7	2.414	0.120	170	82.7	109	82.0	0.053	0.818
	No	39	21.4	29	28.5			50	28.2	39	37.3			35	17.3	24	18.0		

AMR, antimicrobial resistance; GPs, general practitioners; *n* = sample size; NHS, National Health Service; X^2, Pearson's cumulative test statistic and p-value calculated between those who were and were not aware of the campaign annually
